# Genomic and Physiological Traits of the Marine Bacterium *Alcaligenes aquatilis* QD168 Isolated From Quintero Bay, Central Chile, Reveal a Robust Adaptive Response to Environmental Stressors

**DOI:** 10.3389/fmicb.2019.00528

**Published:** 2019-04-05

**Authors:** Roberto E. Durán, Valentina Méndez, Laura Rodríguez-Castro, Bárbara Barra-Sanhueza, Francisco Salvà-Serra, Edward R. B. Moore, Eduardo Castro-Nallar, Michael Seeger

**Affiliations:** ^1^Laboratorio de Microbiología Molecular y Biotecnología Ambiental, Departamento de Química – Centro de Biotecnología, Universidad Técnica Federico Santa María, Valparaíso, Chile; ^2^Department of Infectious Diseases, Institute for Biomedicine, Sahlgrenska Academy, University of Gothenburg, Gothenburg, Sweden; ^3^Culture Collection University of Gothenburg, Sahlgrenska University Hospital, Gothenburg, Sweden; ^4^Centre for Antibiotic Resistance Research, University of Gothenburg, Gothenburg, Sweden; ^5^Microbiology, Department of Biology, University of the Balearic Islands, Palma de Mallorca, Spain; ^6^Center for Bioinformatics and Integrative Biology, Facultad de Ciencias de la Vida, Universidad Andrés Bello, Santiago, Chile

**Keywords:** *Alcaligenes aquatilis*, *Alcaligenes*, aromatic catabolism, benzene, oxidative stress, osmotolerance, phylogenomics, Quintero Bay

## Abstract

*Alcaligenes aquatilis* QD168 is a marine, aromatic hydrocarbon-degrading bacterium, isolated from an oil-polluted sediment of Quintero Bay, an industrial-coastal zone that has been chronically impacted by diverse pollutants. The aims of this study were to characterize the phylogenomic positions of *Alcaligenes* spp. and to characterize the genetic determinants and the physiological response of *A. aquatilis* QD168 to model environmental stressors (benzene, oxidizing agents, and salt). Phylogenomic analyses, using 35 housekeeping genes, clustered *A. aquatilis* QD168 with four other strains of *Alcaligenes* spp. (*A. aquatilis* BU33N, *A. faecalis* JQ135, *A. faecalis* UBA3227, and *A. faecalis* UBA7629). Genomic sequence analyses of *A. aquatilis* QD168 with 25 *Alcaligenes* spp., using ANIb, indicated that *A. aquatilis* BU33N is the closest related strain, with 96.8% ANIb similarity. Strain QD168 harbors 95 genes encoding proteins of seven central catabolic pathways, as well as sixteen peripheral catabolic pathways/reactions for aromatic compounds. *A. aquatilis* QD168 was able to grow on 3-hydroxybenzoate, 4-hydroxybenzoate, benzoate, benzene, 3-hydroxycinnamate, cinnamate, anthranilate, benzamide, 4-aminobenzoate, nicotinate, toluene, biphenyl and tryptophan, as sole carbon or nitrogen source. Benzene degradation was further analyzed by growth, metabolite identification and gene expression analyses. Benzene strongly induced the expression of the genes encoding phenol hydroxylase (*dmpP*) and catechol 1,2-dioxygenase (*catA*). Additionally, 30 genes encoding transcriptional regulators, scavenging enzymes, oxidative damage repair systems and isozymes involved in oxidative stress response were identified. Oxidative stress response of strain QD168 to hydrogen peroxide and paraquat was characterized, demonstrating that *A. aquatilis* QD168 is notably more resistant to paraquat than to H_2_O_2_. Genetic determinants (47 genes) for osmoprotective responses were identified, correlating with observed high halotolerance by strain QD168. The physiological adaptation of *A. aquatilis* QD168 to environmental stressors such as pollutants, oxidative stress and salinity may be exploited for bioremediation of oil-polluted saline sites.

## Introduction

The *Alcaligenes* genus, belonging to the *Alcaligenaceae* family of the order *Burkholderiales* (Garrity et al., 2005), was first described in 1911 during the study of a bacterium isolated from a fecal sample and identified as *A. faecalis* (Kühnemann, 1911). Strains of *Alcaligenes* spp. have been isolated from diverse environments, such as water (Regar et al., 2016), agricultural soil (Liu et al., 2016), crude oil-polluted sites (Singha et al., 2017), industrial processes (Qiu et al., 2017; Basharat et al., 2018), insects (Quiroz-Castañeda et al., 2015) and human clinical samples (Winslow et al., 1920; Al Laham et al., 2017). Strains of *Alcaligenes* spp. have been used for biotechnological applications, such as bioremediation and biocontrol. Strains of *Alcaligenes* spp. are capable of degrading a wide range of xenobiotics, including crude-oil (*Alcaligenes* spp.), polyaromatic hydrocarbons (PHAs) (*A. faecalis* BDB4), pesticides (*Alcaligenes* sp. EGD-AK7), dyes (*A. faecalis* subsp. *phenolicus* MB207), 4-hydroxyacetophenone (*Alcaligenes* sp. 4HAP) and phenols (*A. faecalis* subsp. *phenolicus* DSM 16503^T^ and *A. faecalis* JF339228) (Kiyohara et al., 1982; Hopper and Kaderbhai, 1999; Rehfuss and Urban, 2005; Adebusoye et al., 2007; Kumar et al., 2013; Sagarkar et al., 2014; Regar et al., 2016; Singha et al., 2017; Basharat et al., 2018).

Whole-genome sequencing and genome mining are useful for characterizing bacteria and to discover their biotechnological capabilities. A comparative genomic analysis of *A. faecalis* subsp. *phenolicus* MB207 and 10 other strains of *Alcaligenes* spp. identified biosynthetic clusters for butyrolactone, ectoine, resorcinol, terpene, non-ribosomal peptides and polyketide synthases (Basharat et al., 2018). Strain MB207 is able to degrade azo dyes and possesses tolerance to heavy metals, although, its genomic and physiological stress determinants have not been analyzed. Currently, four complete genome sequences are publicly available for this genus, including those of *A. faecalis* ZD02 (Ju et al., 2016), *A. faecalis* DSM 30030^T^ (Genome accession: GCA_002443155.1), *A. faecalis* JQ135 (Zhang et al., 2018) and *A. faecalis* P156 (Genome accession: GCA_001641975.2), but no whole-genome bioprospecting analyses have been performed previously for any of these strains.

Marine ecosystems are characterized by a wide range of multiple stressors, such as salinity, hypoxia, low and high temperatures, acidic pH, ultraviolet radiation, pollution and scarcity of nutrients. Microorganisms from these locations are well adapted to such environmental pressures. Marine ecosystems are valuable sources for pollutant-degrading and stress-resistant bacterial strains. Although isolates of marine *Alcaligenes* spp. have been described less frequently than their soil counterparts, degradation capabilities and biotechnological applications of marine and coastal strains have been reported. *Alcaligenes* sp. strains ASW3 and ASS-1 were isolated from crude oil-polluted seawater and were applied in an immobilized bacterial consortium for biodegradation of crude-oil (Chen et al., 2017). Mercury-resistant strains of *A. faecalis* were isolated from various locations along the Indian Coast, exhibiting capabilities for cadmium and lead detoxification (De and Ramaiah, 2007).

Quintero Bay is located in an industrial-coastal zone in Valparaíso Region, Central Chile, which has been subjected to extensive and long-term pollution. Quintero is a highly industrialized area for its small population. A crude-oil harbor, copper refinery, thermoelectric power plant, liquefied gas plant and chemical industries are located in this sector. Oil-spills have been documented in this bay several times during the last two decades (Palma-Fleming et al., 2008; Piraino et al., 2017).

Monoaromatic compounds, such as benzene, toluene, ethylbenzene and the three-xylene isomers, commonly known as BTEX, are often present in industrial and crude oil-polluted sites (Fuentes et al., 2014). Benzene is the most toxic and hazardous BTEX compound, being the only carcinogenic pollutant of this group. Marine bacterial strains of species of *Bacillus, Comamonas, Exiguobacterium, Marinobacterium, Pseudomonas* and *Rhodococcus* genera that are capable of degrading BTEX hydrocarbons have been described (Wang et al., 2008; Jiang et al., 2015; Baek et al., 2018). However, information on the genes and catabolism of BTEX compounds by strains of *Alcaligenes* spp. is limited.

Bacterial communities are able to thrive in polluted environments despite the harsh conditions encountered (Michas et al., 2017; Ahmed et al., 2018). Isolation and characterization of multistress-tolerant bacteria from severe environments, lead to potential discovery of novel strains for the clean-up of polluted sites (Head et al., 2006; Fuentes et al., 2014; Orellana et al., 2018). Diverse bacteria, including extremophiles, have been used as biocatalysts for bioremediation of soils and water contaminated with persistent organic pollutants and heavy metals (Harayama et al., 2004; Saavedra et al., 2010; Rojas et al., 2011; Fuentes et al., 2016; Méndez et al., 2017; Orellana et al., 2018).

*A. aquatilis* QD168 was isolated from crude oil-polluted coastal sediments of the industrial zone of Quintero Bay, Central Chile (Durán et al., 2019). The aims of this study were to elucidate the phylogenomic positions of strains of *Alcaligenes* spp., and to characterize the genetic determinants and the physiological responses to model environmental stressors (benzene, oxidizing agents, and salt) of the novel hydrocarbonoclastic marine bacterium *A. aquatilis* QD168. To our knowledge, this is the first phylogenomic study reported for *Alcaligenes* spp. which included 26 strains from diverse environments. Additionally, this is the first genomic characterization of an *A. aquatilis* strain that reveals the genetic potential for aromatic catabolism and physiological responses to endure diverse environmental stresses.

## Materials and Methods

### Chemicals

Benzoate, 3-hydroxybenzoate, 4-hydroxybenzoate, cinnamate, 3-hydroxycinnamate, salicylate, nicotinate, anthranilate, 4-aminobenzoate, 2-aminophenol, benzamide, simazine, tryptophan, and methyl viologen dichloride hydrate (paraquat, PQ) were obtained from Sigma-Aldrich (St. Louis, MO, United States). Benzene (>99.7% purity), toluene, biphenyl, naphthalene, phenanthrene, anthracene, fluorene, and 30% (w v^-1^) hydrogen peroxide (H_2_O_2_) were purchased from Merck (Darmstadt, Germany).

### Culture Enrichment and Isolation of *A. aquatilis* QD168

*A. aquatilis* QD168 was isolated by selective enrichment. 10 g (wet weight) of polluted marine sediment from Quintero Bay, Chile (32°45′49.5″S 71°29′13.5″W) and 90 mL of 0.85% (w v^-1^) NaCl were mixed in a Heidolph blender at high speed for 2 h at room temperature. Bushnell-Haas (BH) mineral medium (1 g L^-1^ KH_2_PO_4_, 1 g L^-1^ K_2_HPO_4_, 1 g L^-1^ NH_4_NO_3_, 0.2 g L^-1^ MgSO_4_, g L^-1^ CaCl_2_ 0.02, 0.05 g L^-1^ FeCl_3_ [pH 7.0]) (Bushnell and Haas, 1941) supplemented with 1% v v^-1^ commercial diesel as sole carbon source, was used for enrichment. Specifically, 45 mL of Bushnell-Haas broth with artificial seawater (23.47 g L^-1^ NaCl, 3.92 g L^-1^ Na_2_SO_4_, 10.64 g L^-1^ MgCl_2_ × 6H_2_O, 1.457 g L^-1^ CaCl_2_ × 2H_2_O, 0.192 g L^-1^ NaHCO_3_, 0.664 g L^-1^ KCl, 0.096 g L^-1^ KBr, 0.026 g L^-1^ H_3_BO_3_, 0.0404 g L^-1^ SrCl_2_ × 6H_2_O, 0.003 g L^-1^ NaF) (BH-saline), supplemented with 200 μg mL^-1^ cycloheximide, in order to inhibit eukaryotic cell growth, in a 250 mL Erlenmeyer flask, was inoculated with 5 mL of supernatant. The enrichment was obtained by 10 consecutive subcultures (1-week intervals) at 20°C with rotary shaking at 180 rpm. The isolation of hydrocarbon degrading bacteria was carried out by serial dilutions. *A. aquatilis* QD168 was obtained by spread-plating on BH agar, supplemented with artificial sea water (ASW) and 200 μg mL^-1^ cycloheximide and subsequently purified by streak-plating. The BH agar plate contained a paper filter with diesel in the lid as the sole carbon source and was incubated at 20°C. Bacterial isolates were stored in 15% (v v^-1^) glycerol at -80°C.

### *Alcaligenes* Genomic Dataset

All the genome sequences of the genus *Alcaligenes* that were available in GenBank in September 2018 (Accession numbers in Supplementary Table S1, including a status of complete, scaffolds, or contigs). The resulting 26 genomes were organized into 24 described species plus 2 unclassified at the species level. All the genome sequences (including the *A. aquatilis* strain QD168 from this study), were re-annotated using a combination of *ab initio* and similarity methods as implemented in Prokka version 1.10 (Seemann, 2014) and NCBI Prokaryotic Genome Annotation Pipeline version 4.6 (Tatusova et al., 2016).

### Average Nucleotide Identity Analysis

Genome-wide nucleotide identity trends were explored in the genome dataset by estimating all-against-all pairwise Average Nucleotide Identity (ANI). The ANIb approach which uses BLAST, was used to align the input sequences as implemented in JSpeciesWS v.3.0.2 (Richter et al., 2016). Heat maps were generated, using pheatmap V 1.0.8 R package (Kolde, 2015). Genomic clusters were defined, using hierarchical clustering (method: average), based on a 95% ANI threshold, which represents a conservative boundary for species level classification (Richter and Rosselló-Móra, 2009).

### Multi-Locus Sequence Analysis (MLSA)

Twenty-eight phylogenetic gene markers implemented in AMPHORA2 were used (*dnaG, frr, infC, nusA, pgk, pyrG, rplB, rplC, rplD, rplE, rplF, rplM, rplN, rplP, rplS, rplT, rpoD, rpmA, rpsB, rpsC, rpsE, rpsI, rpsJ, rpsK, rpsM, rpsS, smpB*, and *tsf*) (Wu and Scott, 2012), as well as seven other genetic markers (*atpD, gltA, gyrB, nirK, pnp, recA*, and *thrC*), from the data set. All nucleotide sequences were aligned, using MAFFT version 7.407 (Katoh and Standley, 2013). The alignments were manually trimmed, using the alignment editor AliView version 1.24 (Larsson, 2014) and concatenated, using the Bio.Nexus module, as implemented in Biopython version 1.72 (Cock et al., 2009). The best partitioning scheme was identified, using the program PartitionFinder version 2.1.1 (Lanfear et al., 2017). A distribution of probable trees was obtained by Bayesian Inference as implemented in MrBayes 3.2.6 (Ronquist et al., 2012). Two separate runs of 20 million generations were executed (four chains each run; sampling every 1,000 generations). Visualization and editing of phylogenetic trees were performed, using the FigTree v. 1.4.2 software^1^.

### Circular *A. aquatilis* QD168 Genome Representation

CGview version 2 (Stothard and Wishart, 2005) was used to generate a circular map of the *A. aquatilis* QD168 genome. Genes were classified according to the Clusters of Orthologous Groups (COG) nomenclature (Galperin et al., 2015), using the eggNOG database version 4.5.1 (Huerta-Cepas et al., 2016).

### Orthologous Gene Search

Previously identified coding sequences were submitted to a local alignment, using the BLASTP and TBLASTN tools of BLAST 2.7.1+ software (Camacho et al., 2009), against a database of enzymes, transcriptional regulators and transporters involved in aromatic compound catabolism, oxidative stress and osmotic stress response reported in UniProtKB-Swissprot (Bateman et al., 2015). An e-value cutoff <10^-10^, identity >30% and coverage >50% were used to filter the outcome. Critical residues and domains for protein function were identified by DELTA-BLAST (Camacho et al., 2009).

### Phylogenetic Analysis of OxyR and SoxR Regulators

OxyR and SoxR amino acid sequences with experimental evidence were retrieved from UniProtKB Swiss-Prot/TrEMBL database (Bateman et al., 2015). All nucleotide sequences were aligned, using MAFFT version 7.407 (Katoh and Standley, 2013). The alignments were manually trimmed, using the alignment editor AliView version 1.24 (Larsson, 2014). The best partitioning scheme was identified, using the program PartitionFinder version 2.1.1 (Lanfear et al., 2017). A distribution of probable trees was obtained, by Bayesian Inference as implemented in MrBayes 3.2.6 (Ronquist et al., 2012). Two separate runs of one million generations were executed (two chains each run; sampling every 1,000 generations). Visualization and editing of phylogenetic trees were performed using the FigTree v. 1.4.2 software (see footnote 1).

### Growth of *A. aquatilis* QD168 on Aromatic Compounds

To assess catabolic potential of *A. aquatilis* QD168, growth assays were performed in M9 minimal medium (6.78 g L^-1^ Na_2_HPO_4_, 3 g L^-1^ KH_2_PO_4_, 0.5 g L^-1^ NaCl, 1 g L^-1^ NH_4_Cl) with trace solutions [154 mg L^-1^ MgSO_4_⋅7H_2_O, 13.4 mg L^-1^ MgCl_2_⋅6H_2_O, 11.9 mg L^-1^ FeSO_4_⋅7H_2_O, 2.5 mg L^-1^ CaCO_3_, 1.8 mg L^-1^ ZnSO_4_⋅7H_2_O, 1.4 mg L^-1^ MnSO_4_⋅H_2_O, 0.35 mg L^-1^ CoCl_2_⋅6H_2_O, 0.3 mg L^-1^ CuSO_4_⋅5H_2_O, 0.075 mg L^-1^ H_3_BO_3_, and 0.0024% (v v^-1^) HCl] (Overwin et al., 2012; Romero-Silva et al., 2013; Agulló et al., 2017) or the nitrogen free M8 minimal medium (6.78 g L^-1^ Na_2_HPO_4_, 3 g L^-1^ KH_2_PO_4_, 0.5 g L^-1^ NaCl) with trace solutions supplemented with 1.75% (w v^-1^) NaCl, and an aromatic compound as sole carbon or nitrogen source.

Protocatechuate (1.33 mM), catechol (1.33 mM), gentisate (1.33 mM), salicylate (1.14 mM), benzoate (1.14 mM), phenol (1.33 mM), 3-hydroxybenzoate (1.14 mM), 4-hydroxybenzoate (1.14 mM), 3-toluate (1.00 mM), cinnamate (0.89 mM), 3-hydroxycinnamate (0.89 mM), benzene (1.33 mM), phenanthrene (0.57 mM), fluorene (0.62 mM) and anthracene (0.57 mM) were tested as sole carbon source for bacterial growth. Anthranilate (1.14 mM), tryptophan (0.72 mM), nicotinate (1.33 mM), 2-aminophenol (1.33 mM), 4-aminobenzoate (1.14 mM), benzamide (1.14 mM), and simazine (1.14 mM) were used in M8 minimal medium as sole carbon and nitrogen source for growth. Bacterial growth was determined using a 2-mL squared 96-well plate by measuring turbidity (600 nm), and using closed 15-mL glass tubes by colony-forming units (CFU) (Méndez et al., 2011). Values were calculated as the mean ± SD of the results of, at least, three independent experiments.

### Growth of *A. aquatilis* QD168 on Benzene

*A. aquatilis* QD168 was cultured in M9 minimal medium with benzene (5 mM) as sole carbon source. For this, cells were previously adapted with successive passages in M9 medium with 0.5, 2.0, and 5.0 mM benzene as carbon source. After adaptation, cells were cultured at 200 rpm and 30°C in M9 medium with 5 mM benzene as sole carbon source. To avoid benzene volatilization, 250-mL glass flasks closed using PTFE/silicone-screw caps were used. Growth was monitored by measuring turbidity at 600 nm until 120 h. Values were calculated as the mean ± SD of results of, at least, three independent experiments.

### Benzene Degradation Assay

Resting cells (turbidity_600 nm_ ∼ 8.0) were incubated in sodium phosphate buffer (50 mM, pH 7.2) with benzene (5 mM). Aliquots of cell suspensions were taken at different incubation times and centrifuged (10,000 × *g* for 10 min). Assays with boiled cells and without cells were used as controls. Resting cell assays were performed in triplicate.

### Intermediate Metabolites Identification

For benzene and phenol quantification, an organic extraction was performed of samples grown in benzene with one volume of ethyl acetate. The mixture was centrifuged at 10,000 × *g* for 5 min. The organic fraction was used for quantification by reverse phase chromatography with a Jasco liquid chromatograph equipped with a diode array detector and a Whatman C-18 column (25 cm by 4.6 mm ID) (Chirino et al., 2013). Methanol:acetonitrile (30:70) was used as the mobile phase with a flux of 1 mL min^-1^.

### *A. aquatilis* QD168 Susceptibility to Oxidizing Agents

*A. aquatilis* QD168 was grown until exponential phase in M9 minimal medium with succinate as sole carbon and energy source. 100-μL of the culture were spread on Petri dishes. 6-mm diffusion disks containing 15 μL of hydrogen peroxide (H_2_O_2_; 1, 5, 10, and 20 mM) or paraquat (PQ; 1, 20, 100, and 500 mM) were deposited on the plates. Growth inhibition diameters were measured after 24 h incubation at 30°C (Sandoval et al., 2011). Values were calculated as the mean ± SD of results of, at least, three independent experiments.

### *A. aquatilis* QD168 Survival After Exposure to Oxidizing Agents

*A. aquatilis* QD168 was grown until exponential phase in M9 minimal medium with succinate as carbon source and washed three times with 2% (w v^-1^) NaCl. Cells were exposed to H_2_O_2_ (0.1, 0.5, 1, and 4 mM) or PQ (25, 50, 100, 200 and 500 mM) during 1 h. Aliquots were taken to determine CFU (Ponce et al., 2011). The percentage of survival was determined as the ratio of CFU of cells exposed to oxidizing agents and CFU of untreated cells. Values were calculated as the mean ± SD of results of, at least, three independent experiments.

### Halotolerance Assays

*A. aquatilis* QD168 was grown until exponential phase (turbidity_600 nm_ ∼ 0.6) in M9 minimal medium with succinate as sole carbon and energy source. 20-μL of the culture was plated on Reasoner’s 2A (R2A) medium with 0, 2, 4, 6, 8, and 10% (w v^-1^) NaCl. The growth was monitored after 24 and 48 h of incubation at 30°C (Gallardo-Cerda et al., 2018). Growth assays to assess halotolerance were performed in three independent experiments.

### Gene Expression Analysis

To analyze the expression of genes involved in benzene catabolism, *A. aquatilis* QD168 was grown until exponential phase in M9 minimal medium with benzene or succinate as sole carbon and energy sources. To analyze the expression of genes involved in oxidative stress response, *A. aquatilis* QD168 was grown until exponential phase in M9 minimal medium with succinate (2 mM) as sole carbon and energy sources, washed three times with 0.85% (w v^-1^) NaCl, and incubated in 1.75% (w v^-1^) NaCl in absence (untreated cells) or presence of H_2_O_2_ (0.5 mM), PQ (50 mM) during 1 h (Ponce et al., 2011).

Bacterial cells were harvested, and RNA was extracted, using QIAGEN RNeasy purification kit (Qiagen; Hilden, Germany), following the manufacturer’s instructions. Contaminant DNA was removed, employing the TURBO DNA-free Kit (Thermo Fisher Scientific; Waltham, MA, United States). RNA quality was verified with the NanoDrop One spectrometer (Thermo Fisher Scientific; Waltham, MA, United States). RNA integrity was checked in a 1% (w v^-1^) agarose gel. Amplification of the 16S rRNA gene by qPCR was performed as control for DNA contamination. cDNA was synthetized with the First Strand cDNA Synthesis Kit (Thermo Fisher Scientific; Waltham, MA, United States). The qRT-PCRs were carried out with the KAPA SYBR FAST qPCR Master Mix Kit (Kapa Biosystems; Boston, MA, United States), following the manufacturer’s instructions. The genes 16S rRNA and *ftsZ* were employed as reference genes. The results were analyzed, using Hellemans method (Hellemans et al., 2007). Succinate-grown and untreated cells were used as control. Negative and positive controls were included in each RT-PCR assay. At least three independent RNA samples were analyzed at each condition and two independent RT-PCR reactions for each sample were done to assess reproducibility. Genes and primers employed are listed in Table 1.

**Table 1:** Oligonucleotides designed and used in this study.

Gene	Primer	Sequence (5′–3′)
16S rRNA	QD168_16SrRNA F	GCTAGTAATCGCGGATCAGAAT
	QD168_16SrRNA R	GGCTACCTACTTCTGGTGAAAC
*ftsZ*	QD168_ftsZ F	CGTTCACTCTCTGATCGTTGTA
	QD168_ftsZ R	CAGGCATTGTGCAAGACATC
*dmpP*	QD168_dmpP F	GCACGTACGCAAGGTAGAA
	QD168_dmpP R	GCACAAAGAACTGCCCATAAG
*catA*	QD168_catA F	GATGGTCCGTTTGCAGAGAT
	QD168_catA R	GGACGATTCACGATCTGGTT
*catE*	QD168_catE F	GTCCAGGATCTGGAACAAACA
	QD168_catE R	CGGGATCACGCAAGAAGAA
*ahpC1*	QD168_ahpC_1 F	GTAATAGCTGGGGCGTCTTG
	QD168_ahpC_1 R	TTGGCGGTATAACCCAGTTC
*ahpC2*	QD168_ahpC_2 F	CCATGATCGGTGACCCTACT
	QD168_ahpC_2 R	CGCAAAGCGATACCTTCTTC
*katE*	QD168_katE F	ATTTCGACCACGAGCGTATC
	QD168_katE R	GGCCTGCGTACATTCAAAAT
*sodC1*	QD168_sodC1 F	ACTGGCGGACGTCATTATTC
	QD168_sodC1 R	CATGTACACCGGGTTTGAGA
oxyR	QD168_oxyR_1 F	TGACATTGACCGAGCTGAAG
	QD168_oxyR_1 R	CGAAGATGACCACGCCTAAT
*perR*	QD168_perR F	TGGCTACGAGCCTTTGAATC
	QD168_perR R	TTCCAGGCTCTTGACCTGAT
*soxR1*	QD168_soxR_1 F	ATAGGCTGTGGATGCTTGTC
	QD168_soxR_1 R	GGTGCTCTTCTGCTTCAGTAA
*soxR2*	QD168_soxR_2 F	TCCTTTGGAGGAAATTCACG
	QD168_soxR_2 R	CTGCTCAATCCGATCATTCA
*fumC*	QD168_fumC F	CTGGAACCTGTCATGGAGAAA
	QD168_fumC R	GCCTTGTCGTAACCGATGT

### Statistical Analyses

Statistical analyses were performed using one-way ANOVA and Tukey HSD test to assess differences in mean values from each experiment. Differences are significant at α = 0.05.

## Results

### Phylogenomic Analysis of *Alcaligenes* Genus

**FIGURE 1:**
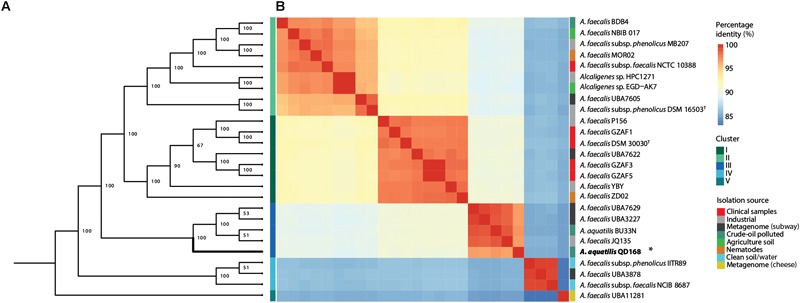
Phylogenetic analysis of representative *Alcaligenes* spp. strains including *A. aquatilis* QD168. **(A)** MLSA-based clustering of 26 strains of *Alcaligenes* spp. based on 35 housekeeping genes. Mid-rooted phylogeny showed five distinctive clades. **(B)** ANIb analysis of 26 strains of *Alcaligenes* spp. The first column indicates 95% ANI clustering (75% alignment). Isolation source is indicated in the last column. *A. aquatilis* QD168 belongs to cluster III.

To investigate the phylogenomic relationships of strains of *Alcaligenes* spp., a more comprehensive set of housekeeping gene markers was obtained from strain QD168 and 25 publicly available genomes of *Alcaligenes* spp. (Supplementary Table S1), to perform a multi-locus sequence analysis (MLSA). AMPHORA2 was used to evaluate the presence of single copy genes encoding ribosomal proteins, as well as some other classic bacterial marker genes, in all the genomes evaluated. This analysis revealed 28 genetic markers and seven additional housekeeping genes were manually incorporated to obtain a total of 35 housekeeping genes, including the *gyrB* and *nirK* genes, which have been assessed in the taxonomic identification of *A. pakistanensis* (Abbas et al., 2015). The following genome sequences available at GenBank were not included in this, and the following analyses: *A. faecalis* GZAF2 (accession no.: GCA_002119985.1) and *A. faecalis* GZAF4 (GCA_002120025.1), due to the absence of relevant genetic markers (*gyrB, dnaG* or *nirK)* selected for the MLSA. Figure 1A, shows the phylogenetic relationships between strains of *Alcaligenes* spp. Interestingly, strains of *Alcaligenes* spp. clustered in five distinct clades, which did not correlate with their previous species identification. *A. aquatilis* QD168 clustered in close proximity with four other *Alcaligenes* strains: *A. aquatilis* BU33N, the only other *A. aquatilis* genome available (Genome accession: GCA_0030765151.1); *A. faecalis* JQ135, a 5-hydroxypicolinic acid and nicotinic acid degrader (Qiu et al., 2017; Zhang et al., 2018); *A. faecalis* UBA3227 and *A. faecalis* UBA7629, whose genomes were obtained from subway metagenomes (Figure 1A).

To further study the genome-genome relationships between strains of *Alcaligenes* spp., an average nucleotide identity based on BLAST (ANIb) analysis was performed with *A. aquatilis* QD168 and 25 strains of *Alcaligenes* spp. from the dataset (Figure 1B). The most similar strain to *A. aquatilis* QD168 is *A. aquatilis* BU33N (96.8%), suggesting that both strains belong to the same species. *A. aquatilis* BU33N was isolated from a petroleum-polluted sediment from Tunisia (BioProject: PRJNA386470). Using the proposed boundary for species delineation (ANIb value >95%, Richter and Rosselló-Móra (2009)) the 26 strains of *Alcaligenes* spp. were grouped in five genomic clusters correlating with the information obtained by the MLSA analysis (Figure 1B). The cluster I comprised eight strains (*A. faecalis* P156, *A. faecalis* DSM 30030^T^, *A. faecalis* GZAF1, *A. faecalis* UBA7622, *A. faecalis* GZAF3, *A. faecalis* GZAF5, *A. faecalis* ZD02, and *A. faecalis* YBY) containing the type strain for *A. faecalis* species (DSM 30030^T^) and including most of the clinical isolates from the dataset. The cluster II, which belonged to a wide range of isolation source environments, included nine strains (*A. faecalis* BDB4, *A. faecalis* NBIB-017, *A. faecalis* subsp. *phenolicus* MB207, *A. faecalis* MOR02, *A. faecalis* subsp. *phenolicus* 10388, *Alcaligenes* sp. HPC1271, *Alcaligenes* sp. EGD-AK7, *A. faecalis* UBA7605 and *A. faecalis* subsp. *phenolicus* DSM 16503^T^) containing the type strain for the subsp. *phenolicus* of *A. faecalis* species (DSM 16503^T^). The cluster III included *A. aquatilis* QD168, and four other strains (*A. faecalis* UBA3227, *A. faecalis* UBA7629, *A. aquatilis* BU33N and *A. faecalis* JQ135). Three of these strains were isolated from polluted environments, and the genomes of two strains were retrieved from subway metagenomes. The cluster IV, contained three strains (*A. faecalis* subsp. *phenolicus* IITR89, *A. faecalis* UBA3878 and *A. faecalis* subsp. *faecalis* NCIB 8687); two members were isolated from clean soil or water sources. Finally, cluster V, consisted of a singleton containing *A. faecalis* UBA11281 (Figure 1B). The genome of strain UBA11281 was retrieved from metagenome data of a cheese sample.

### Genome Assembly and Clusters of Orthologous Groups Analyses

**FIGURE 2:**
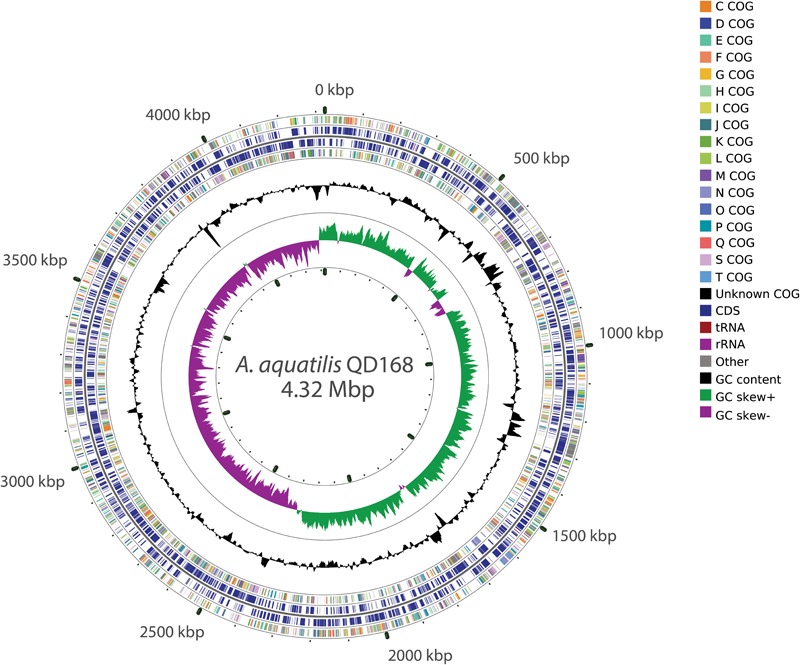
Schematic representation of the genome of *A. aquatilis* QD168. *Alcaligenes aquatilis* QD168 genome has a size of 4,323,879 bp and contains 3,892 coding sequences, 9 rRNA and 58 tRNA. Rings from inside to outside: (1) G + C skew; (2) G + C content; (3) QD168 genes colored by functional COG class on the reverse strand; (4) predicted QD168 genes on the reverse strand; (5) predicted QD168 genes on the forward strand; (6) QD168 genes colored by functional COG class on the forward strand. Gene functions were annotated based on COG categories. The whole genome map was generated using CGView.

**FIGURE 3:**
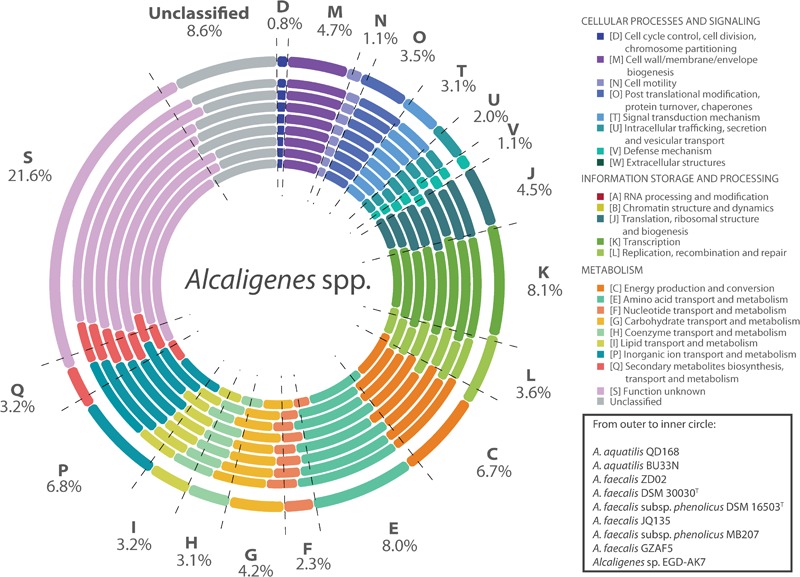
Cluster of orthologous groups comparison of *A. aquatilis* QD168 and representative *Alcaligenes* strains. Cluster of Orthologs (COG) classification of eight *Alcaligenes* spp. strains. For each COG entry the average percentage of hits among 26 *Alcaligenes* spp. strains has been indicated by values and dashed lines. From outer to inner circle: *A. aquatilis* QD168, *A. aquatilis* BU33N *A. faecalis* ZD02, *A. faecalis* DSM 30030^T^ *A. faecalis* subsp. *phenolicus* DSM 16503^T^ *A. faecalis* JQ135, *A. faecalis* subsp. *phenolicus* MB207, *A. faecalis* GZAF5, *Alcaligenes* sp. EGD-AK7.

The genomes of strains of *Alcaligenes* spp. included in this study have a size range between 3.68 and 4.44 Mbp (average 4.14 Mbp), and possess a GC content between 55.4 and 57.6% (average 56.6%) (Supplementary Table S1). *A. aquatilis* QD168 has a genome size of 4,323,879 bp, with a GC content of 56.4%, assembled in one circular chromosome. The genome of strain QD168 contains 3,892 coding sequences (CDS) (Durán et al., 2019). The functions of 3,557 genes of *A. aquatilis* QD168 were annotated, based on Clusters of Orthologous Groups (COG) categories (Galperin et al., 2015), corresponding to 91.4% of total CDS (Figure 2). The three most abundant categories were: unknown function (S; 23.67%), transcription (K; 7.95%), and amino acid transport and metabolism (E; 7.40%). A COG comparison analysis was performed between the 26 strains of *Alcaligenes* spp. The functions of the 26 predicted proteomes, obtained by Prokka annotation of the genomic dataset, were classified based on COG categories. Figure 3 shows the percentage of each COG category for the proteomes of eight specific *Alcaligenes* members. From outer to inner circle: *A. aquatilis* QD168; *A. aquatilis* BU33N; the reference genome for the species *A. faecalis, A. faecalis* ZD02; the type strain of *A. faecalis* species *A. faecalis* DSM 30030^T^, the type strain *A. faecalis* subsp. *phenolicus* DSM 16503^T^; the nicotinic acid-degrader *A. faecalis* JQ135; the azo dye-degrader *A. faecalis* subsp. *phenolicus* MB207; the clinical human isolate *A. faecalis* GZAF5, and the atrazine-degrader *Alcaligenes* sp. EGD-AK7 (Supplementary Table S2). In general, the distribution of COG categories among the genus is highly similar, obtaining the highest average percentages for (S) function unknown (21.6%), (K) transcription (8.1%), and (E) amino acid transport and metabolism (8.0%) categories (Figure 3, dashed lines). *A. aquatilis* QD168 possessed a higher percentage of orthologous genes compared to the average of all the strains of *Alcaligenes* spp. included in this study on the categories functions: (S) function unknown (S: 23.27%) in comparison to 21.6 ± 1.20%; (L) replication, recombination and repair (L: 5.11%) compared to 3.62 ± 0.47% and the category (U) intracellular trafficking, secretion, and vesicular transport (U: 2.68%) in comparison to 2.02 ± 0.36% (Figure 3).

### Metabolic Potential for Aromatic Compounds Degradation of Strain QD168

**FIGURE 4:**
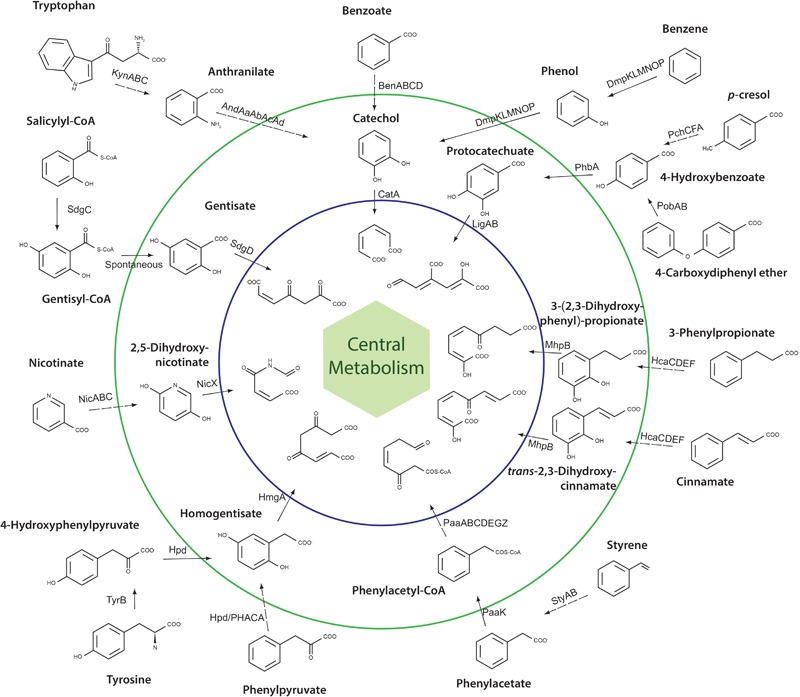
Schematic representation of all central aromatic catabolic pathways and peripheral aromatic catabolic pathways/reactions present in *A. aquatilis* QD168. The inner circle (blue) includes ring-cleavage product structures of central aromatic catabolic pathways. The outer circle (green) includes the structure of aryl-CoA and dihydroxylated ring-cleavage intermediates. Dashed lines indicate multiple steps.

In order to predict the catabolic potential for aromatic compounds of *A. aquatilis* QD168, a genomic search for genes of aromatic catabolic pathways was performed. The genes of seven aromatic central pathways, and sixteen peripheral pathways/reactions were identified in the QD168 genome. Central pathways included the catechol (*cat*), protocatechuate (*lig*), homogentisate (*hmg*), gentisate (*nag*), phenylacetyl-CoA (*paa*), 3-(2,3-dihydroxyphenyl)-propionate (*mhp*), and 2,5-dihydroxynicotinate (*nic*) (Figure 4).

The genes encoding the catechol branch of the β-ketoadipate pathway were found clustered (*catRBAIJDC*), whereas the genes encoding the protocatechuate branch (*pca*; *ortho*-cleavage pathway) were not present in strain QD168. The *cat* gene products of *A. aquatilis* QD168 were highly similar to proteins of the catechol branch that has been characterized mainly in bacteria of *Acinetobacter* and *Pseudomonas* genera (Supplementary Table S3). Interestingly, strain QD168 possessed the genes encoding the peripheral reactions for benzoate (*benMABCD*), benzene and phenol (*dmpRKLMNOP*), anthranilate (*andAaAbAcAd*), and tryptophan (*kynABU*) that converge into catechol as a central intermediate (Figure 4 and Supplementary Table S4). The *ben, dmp*, and *kyn* gene products of strain QD168 were highly similar to the corresponding characterized proteins of bacteria of *Acinetobacter, Burkholderia*, and *Pseudomonas* genera (Supplementary Table S4).

The genes encoding the protocatechuate *meta*-cleavage (*ligCBAIKJ*) were found clustered in the QD168 genome. The *ligCBA* gene products possessed high identity (>58%) to the proteins of *Sphingomonas paucimobilis* SYK-6 (Supplementary Table S3). The *ligIKJ* gene products of strain QD168 possessed high identity (70%) to the proteins of *Comamonas* sp. E6, *Pseudomonas straminea* and *Acidovorax* sp. T1 (Supplementary Table S3). Interestingly, the gene D3M96_07080, encoding a 4-hydroxybenzoate transporter (PcaK), was found within this genomic context. The genes encoding peripheral reactions for 4-hydroxybenzoate (*phbA*), *p*-cresol (*pchCFA*), and 4-carboxy-diphenyleter (*pobAB*) that channel into the protocatechuate *meta*-cleavage pathway were identified in the QD168 genome (Supplementary Table S4). The *phbA* and *pobAB* gene products of strain QD168, were highly similar to the PhbA, PobA, and PobB proteins of strains of *Pseudomonas* spp. The *pchCFA* gene products of strain QD168, which encode enzymes for *p*-cresol conversion into 4-hydroxybenzoate and further protocatechuate formation, possessed high identities with the proteins of *P. putida* NCIMB 9869, and were located within the neighborhood of the *lig* genes (protocatechuate central pathway).

The *paa* genes encoding the phenylacetyl-CoA central pathway, were grouped in the *paaABCDEZGI* gene cluster next to the *paaK* gene that encodes the enzyme of the peripheral reaction for phenylacetate degradation into phenylacetyl-CoA. The *paa* gene products of strain QD168 were highly similar to the corresponding proteins of *Escherichia coli* K-12 (Supplementary Table S3). Additionally, the genes encoding enzymes of peripheral reactions for styrene degradation (*styAB*) via phenylacetate that is funneled into phenylacetyl-CoA central pathway, were identified in the QD168 genome (Supplementary Table S4).

Catabolic genes encoding for tyrosine (*tyrB*), phenylpyruvate (*hpd*), and 4-hydroxyphenylpyruvate (*hpd*) degradation via the homogentisate central pathway were identified in the QD168 genome. The gene products possessed >55% identity with the Hpd and TyrB proteins of *Pseudomonas* and *E. coli* strains (Supplementary Table S4). Although the *hmgA, hmgB*, and *hmgC* genes, encoding the proteins of the homogentisate central pathway are located in an operon in Gammaproteobacteria, such as *E. coli* and *P. putida*, the *hmg* genes were found distributed in *A. aquatilis* QD168 genome. Interestingly, the *hmg* genes products of strain QD168 were highly similar to the HmgA and HmgB proteins of *Bordetella* genus (>65% identity) and HmgC of *P. aeruginosa* (47% identity) (Supplementary Table S3).

The *sdgD, nagL*, and *nagK* genes encoding the enzymes of gentisate central pathway, were distributed within the QD168 genome. The enzyme of the peripheral reaction for salicylyl-CoA (SdgC) that is channeled into gentisyl-CoA, and then to the gentisate central pathway, was identified in strain QD168, and possessed 40% identity to the characterized SdgC protein of *Streptomyces* sp. WA46 (Supplementary Table S4).

The *mhpBCDFE* genes encoding the 3-(2,3-dihydroxyphenyl)-propionate/2,3-dihydroxy-cinnamate central catabolic pathway, were found clustered and located next to the *hcaREFCBC* gene cluster, which encodes the 3-phenylpropionate and cinnamate peripheral pathways (Figure 4 and Supplementary Table S4). The genes encoding the 2,5-dihydroxynicotinate central catabolic pathway (*nicDXF*) in strain QD168, were similar (>43% identity) to the corresponding enzymes of *P. putida* KT2440. Two copies of the *nicDXF* gene cluster, were found in the QD168 genome. The *nicAB* gene products of strain QD168 involved in the degradation of nicotinate into the 2,5-dihydroxynicotinate central catabolic pathway, were also similar (>41% identity) to the NicA and NicB enzymes of *P. putida* KT2440 (Supplementary Table S3).

### Functional Aromatic Degradation Pathways in *A. aquatilis* QD168

To assess the degradation capabilities of strain QD168, growth assays on aromatic compounds as sole carbon or nitrogen source were performed. Twenty-five aromatic compounds were tested. *A. aquatilis* QD168 was able to grow in minimal medium on thirteen aromatic compounds. Strain QD168 was able to grow on 3-hydroxybenzoate, 4-hydroxybenzoate, benzoate, benzene, 3-hydroxycinnamate, cinnamate, anthranilate, benzamide, 4-aminobenzoate, nicotinate, tryptophan, toluene, and biphenyl as sole carbon or nitrogen source (Table 2). No color formation, that may indicate *meta*-cleavage of the aromatic ring, was observed during growth on aromatic compounds.

**Table 2:** Growth of *A. aquatilis* QD168 on aromatic compounds.

Compound	Carbon source (M9 minimal medium)	Carbon and nitrogen source (M8 minimal medium)
Monocyclic Aromatic Hydrocarbons (MAH)		
Benzene	+++	NA
Toluene	++	NA
Polycyclic Aromatic Hydrocarbons (PAH)		
Biphenyl	+	NA
Naphthalene	-	NA
Anthracene	-	NA
Phenanthrene	-	NA
Fluorene	-	NA
Phenols and Benzoates		
Phenol	++	NA
Benzoate	+++	NA
3-Hydroxybenzoate	+	NA
4-Hydroxybenzoate	+++	NA
Salicylate	-	NA
Carboxylic acids		
Cinnamate	+++	NA
3-Hydroxycinnamate	+	NA
Nitrogenated compounds		
Nicotinate	+++	+++
Anthranilate	+	+
4-Aminobenzoate	ND	+++
2-Aminophenol	ND	-
Tryptophan	+	+++
Benzamide	ND	+++
Simazine	ND	-
Others		
3-toluate	-	NA

*-, No growth (turbidity_*600nm*_ < 0.1); +, low growth (turbidity_*600nm*_ = 0.1-0.2); ++, moderate growth (turbidity_*600nm*_ > 0.2-0.3); +++, high growth (turbidity_*600nm*_ > 0.3) after 120 h.*

### Functional Benzene Catabolic Pathway

**FIGURE 5:**
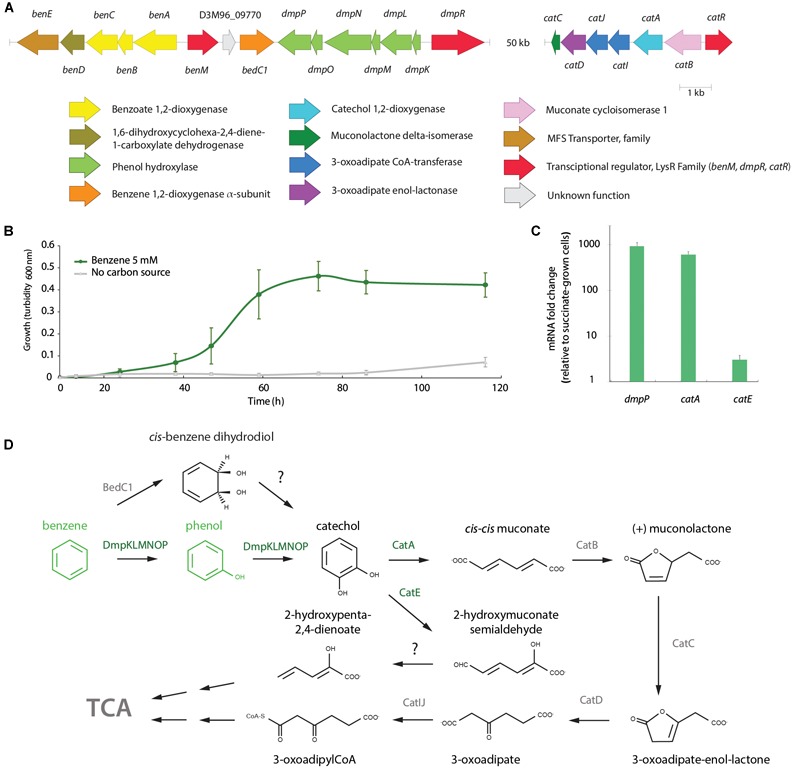
Functional analyses of benzene degradation by *A. aquatilis* QD168. **(A)** Predicted *ben, dmp* and *cat* genes encoding the catechol branch of the β-ketoadipate catabolic pathway and peripheral reactions on *A. aquatilis* QD168 genome. Genes and intergenic regions sizes are on scale **(B)** *A. aquatilis* QD168 growth on benzene (5 mM) as sole carbon and energy sources. Turbidity values were calculated as the mean ± SD of results of, at least, three independent experiments. **(C)** Expression of *dmpP, catA* and *catE* genes during QD168 growth on benzene (5 mM). 16S rRNA and *ftsZ* genes were used as reference genes. At least three independent RNA samples were collected at each condition and two independent RT-PCR reactions for each sample were done to assess reproducibility. **(D)** Proposed benzene degradation pathway of *A. aquatilis* QD168. Enzymes and compounds with experimental data are shown in green.

The degradation of the model aromatic compound benzene was further analyzed due to its wide distribution as part of the highly toxic BTEX compounds and its major environmental concern. *A. aquatilis* QD168 possesses the genes for benzene degradation via phenol into catechol (Figure 4). The *dmpRKLMNOP* gene cluster that encodes the multicomponent phenol hydroxylase was identified in the QD168 genome (Figure 5A). The *benMABCDE* gene cluster was located downstream to the *dmp* cluster. The *benABC* genes encode a multicomponent benzoate 1,2-dioxygenase, and the *benD* gene encodes a *cis-*diol dehydrogenase that are involved in benzoate degradation via catechol (Figure 4). Two coding sequences were identified between the *dmp* and *ben* clusters, the *bedC1* gene that encodes a benzene 1,2-dioxygenase alpha subunit, and the D3M96_09770 gene with unknown function (Figure 5A). However, the *bedA* and *bedB* genes encoding the benzene 1,2-dioxygenase reductase and ferredoxin subunits, respectively, were not found in the QD168 genome. The *cat* genes encoding the catechol *ortho*-cleavage pathway were found in the QD168 genome (Figure 5A), while only the catechol 2,3-dioxygenase encoded by the *catE* gene (D3M96_10490) of the *meta*-cleavage pathway was identified. The *catE* gene product of strain QD168 showed low identity (26% identity) with the catechol 2,3-dioxygenase of *Cupriavidus pinatubonensis* JMP222. *A. aquatilis* QD168 was able to grow in minimal medium in presence of 1.75% (w v^-1^) NaCl and benzene (5 mM) as sole carbon source, attaining a turbidity of ∼ 0.5 at 600 nm after 74 h (Figure 5B). After 60 min incubation of resting cells, benzene was not detected by HPLC, strongly suggesting that strain QD168 is able to degrade benzene. Phenol was identified by HPLC as a metabolic intermediate during benzene degradation. To correlate bioinformatic predictions and functional assays, transcriptional analysis of key catabolic genes for benzene degradation was performed. The transcription of catechol dioxygenase-encoding genes for *meta-* and *ortho-*cleavage (*catA* and *catE*), and the phenol hydroxylase-encoding gene for benzene monooxygenation reaction (*dmpP* gene) was determined. An up-regulation of *dmpP* (>930-fold), *catA* (>610-fold), and *catE* (>3-fold) genes was observed in exponentially growing cells of strain QD168 on benzene, compared to succinate-grown cells (Figure 5C). These results suggest that benzene degradation in strain QD168 occurs mainly via phenol formation as metabolic intermediate and catechol *ortho*-cleavage, by the phenol hydroxylase and the catechol 1,2-dioxygenase enzymes, respectively. Figure 5D illustrates the proposed benzene catabolic pathway in *A. aquatilis* QD168.

### Identification of Oxidative Stress Response Genes and Phylogenetic Analysis of OxyR and SoxR Regulators

**FIGURE 6:**
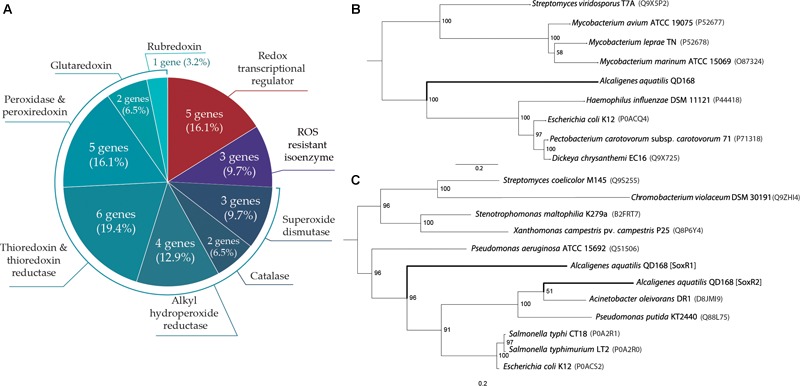
Genetic determinants of *A. aquatilis* QD168 for oxidative stress response and phylogenetic relationships of OxyR and SoxR transcriptional regulators. **(A)** Genetic determinants involved in oxidative stress response identified by genome mining. **(B)** Phylogenetic tree of OxyR protein orthologous: mid-rooted phylogeny showed the clade of QD168 OxyR **(C)** Phylogenetic tree of SoxR orthologous proteins: mid-rooted phylogeny showed five distinctive clades.

Genome analyses indicate that thirty genes of *A. aquatilis* QD168 may participate in scavenging of reactive oxygen species (ROS), resistance to endogenous redox imbalance and regulation of oxidative stress response (Supplementary Table S5). Genes encoding a thioredoxin/thioredoxin reductase system (seven genes), a glutaredoxin/glutathione peroxidase system (four genes), alkyl hydroperoxide reductases (four genes), catalases (two genes), and superoxide dismutases (three genes) were identified in the QD168 genome (Figure 6A). Additionally, five genes encoding transcriptional regulators for oxidative stress response were found. Genes encoding for the SoxR redox-sensitive transcriptional regulator (*soxR1, soxR2*), the H_2_O_2_-sensing transcriptional regulator OxyR (*oxyR*), the transcriptional regulator (*perR*), and the organic hydroperoxide resistance transcriptional regulator OhrR (*ohrR*) were identified.

To further study oxidative stress response in strain QD168, the phylogeny of OxyR, SoxR1 and SoxR2 transcriptional regulators of *A. aquatilis* QD168 was studied (Figure 6). For OxyR (Figure 6B), the amino acid sequence of nine OxyR regulators with experimental evidence in bacteria of *Actinobacteria* and *Gammaproteobacteria* classes were used for bioinformatic analyses. This analysis revealed three distinctive clades, wherein OxyR of *A. aquatilis* QD168 formed a singleton. OxyR orthologs of *Actinobacteria* clustered together, while OxyR orthologs from *Gammaproteobacteria* were grouped in the third clade (Figure 6B). For SoxR1 and SoxR2 analyses (Figure 5C), the amino acid sequence of 10 SoxR transcriptional regulators with experimental evidence of *Gammaproteobacteria, Actinobacteria* (*Streptomyces coelicolor* M145) and *Betaproteobacteria* (*Chromobacterium violaceum* DSM 30191) classes were used. SoxR1 of *A. aquatilis* QD168 formed a singleton ungrouped with other SoxR orthologs. SoxR2 of strain QD168 grouped with *Gammaproteobacteria* orthologs. This phylogenetic clade, consisted of a subgroup of SoxR orthologs of enteric bacteria (*E. coli* K12, *Salmonella typhi* CT18 and *S. typhimurium* LT2), and another subgroup of non-enteric bacteria (*P. putida* KT2440 and *A. oleovorans* DR1) that included SoxR2 ortholog of *A. aquatilis* QD168.

### Response of *A. aquatilis* QD168 to Oxidizing Agents

**FIGURE 7:**
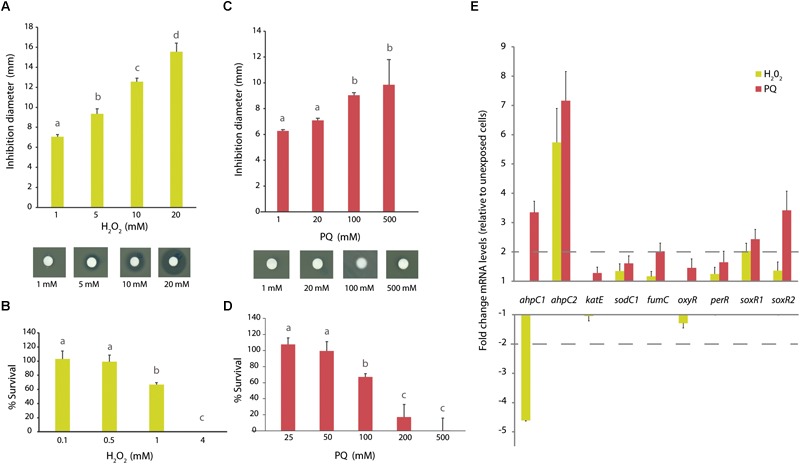
Oxidative stress response of *A. aquatilis* QD168 to H_2_O_2_ and paraquat. **(A)** Growth inhibition diameter of *A. aquatilis* QD168 after 60 min exposure to H_2_O_2_ (1–20 mM). **(B)** Survival of *A. aquatilis* QD168 after 60 min exposure to H_2_O_2_ (0.1–4 mM). **(C)** Growth inhibition diameter of *A. aquatilis* QD168 after 60 min exposure to PQ (1–100 mM). **(D)** Survival of *A. aquatilis* QD168 after 60 min exposure to PQ (25–500 mM). Values for growth inhibition diameter and survival of strain QD168 were calculated as the mean ± SD of results of, at least, three independent experiments. Different letters indicate significant differences between conditions (HSD Tukey test, α = 0.05) **(E)** Expression of oxidative stress genes of *A. aquatilis* QD168 cells after 60 min exposure to H_2_O_2_ (500 μM) and PQ (50 mM). 16S rRNA and *ftsZ* were used as reference genes. At least three independent RNA samples were collected at each condition and two independent RT-PCR reactions for each sample were done to assess reproducibility.

The response of *A. aquatilis* QD168 to H_2_O_2_, and the redox-cycling compound paraquat (PQ) was investigated. To study strain QD168 susceptibility to oxidative stress inducers, cells grown until exponential phase were incubated in presence of H_2_O_2_ or PQ. Strain QD168 showed an increase of the growth inhibition zone in response to increasing concentrations of H_2_O_2_ (1, 5, 10, and 20 mM) (Figure 7A). During strain QD168 cells exposure to 1 and 20 mM PQ, no significant differences were detected. A small increase in the inhibition zone was observed in strain QD168 growth after exposure to higher concentrations of PQ (100 and 500 mM) (Figure 7C).

To study *A. aquatilis* QD168 survival during exposure to oxidizing agents, exponentially growing cells were incubated with H_2_O_2_ or PQ for 1 h. After exposure to 0.1 and 0.5 mM H_2_O_2_, strain QD168 survival was almost 100%, but it significantly decreased at 1 mM H_2_O_2_. After exposure to 4 mM H_2_O_2_, no viable cells were observed (Figure 7B). Interestingly, after exposure to 25 and 50 mM PQ, cell viability was not affected. In contrast, 100 mM PQ caused a significant decrease in cell survival (Figure 7D). After exposure to 200 and 500 mM PQ, cell viability was highly affected, but no significant differences were observed between both concentrations. Overall, these results indicate that *A. aquatilis* QD168 is notably more resistant to PQ than to H_2_O_2_.

To further characterize the bacterial response to oxidizing agents, a gene expression analysis was carried out. For this assay, nine genes were selected: genes encoding the antioxidant enzymes alkyl hydroperoxide reductase (*ahpC1* and *ahpC2*), catalase E (*katE*), superoxide dismutase (*sodC1*), a ROS-resistant isoform of fumarate hydratase (*fumC*), and four transcriptional regulators involved in oxidative stress response (*oxyR, perR, soxR1*, and *soxR2*) (Figure 7E). In response to H_2_O_2_ (0.5 mM), the *ahpC1* gene was down-regulated (>4-fold), while the *ahpC2* gene was up-regulated (>5-fold) compared to control cells. SoxR1 was the only transcriptional regulator whose expression was up-regulated (2-fold) by H_2_O_2_. In response to PQ (50 mM), both genes encoding alkyl hydroperoxide reductase proteins (*ahpC1* and *ahpC2*) displayed an increase of >3- and 7-fold, respectively. An up-regulation (>2-fold) by PQ of the *fumC* gene encoding the enzyme fumarate hydratase C, which is involved in Krebs cycle, was observed. In response to PQ, the genes encoding the transcriptional regulators SoxR1 and SoxR2 were up-regulated >2- and 3-fold, respectively (Figure 7E).

### Osmoprotective Response of *A. aquatilis* QD168

**FIGURE 8:**
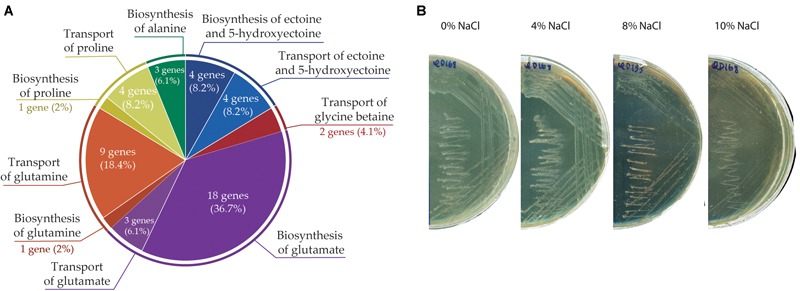
Osmotolerance response of *A. aquatilis* QD168 to salt stress. **(A)** Genetic determinants involved in biosynthesis and transport of osmo-protectant metabolites. **(B)** Salt tolerance of *A. aquatilis* QD168 in R2A medium supplemented with 0 to 10% (w v^-1^) NaCl.

Forty-seven genes that may participate in osmoprotection (synthesis and transport of compatible solutes) were identified in *A. aquatilis* QD168 genome (Supplementary Table S6). Genes involved in the synthesis (*ectABCD*) and transport (*ehuBCDA*) of ectoine/hydroxyectoine, synthesis (18 genes) and transport (4 genes) of glutamate, synthesis (*glnE*) and transport (*glnHPQ* and *bztDCBA*) of glutamine, synthesis (*proA, proBC*, and *argF*) and transport (*putP*) of proline, transport of glycine betaine/choline (*ousVW*), and synthesis (*iscS, sufS*, and *sufE*) of alanine were identified in strain QD168 (Figure 8A). To study the halotolerance of *A. aquatilis* QD168, cells grown in LB medium until exponential phase were plated on R2A medium supplemented with NaCl ranging from 0 to 10% (w v^-1^). Bacterial cell growth was observed at all salt concentration assessed after 48 h of incubation at 30°C (Figure 8B). These results indicate that *A. aquatilis* QD168 is a halotolerant bacterium.

## Discussion

Strains of the *Alcaligenes* spp. have been associated with diverse ecological niches, suggesting genetic diversity and physiological potential to encounter severe conditions, such as the presence of toxic organic compounds, heavy metals, oxidative stress and high salinity.

The phylogenomic analysis, in this study, of strains of *Alcaligenes* spp. revealed five clustering profiles (clusters I–V), which were distributed, in general, according to their isolation sources (Figure 1). The phylogenomic analysis, placing *A. aquatilis* QD168 within the *Alcaligenes* genus, also revealed the same clustering pattern of strains by the MLSA of 35 housekeeping genes and by using ANIb similarities (Konstantinidis and Tiedje, 2005; Richter and Rosselló-Móra, 2009). Genomes corresponding to the *Alcaligenes* genus were highly represented by the species, *A. faecalis*, comprising approximately 84.6% of all genomes of *Alcaligenes* spp. analyzed. However, the presence of, at least, one strain classified as *A. faecalis* in every phylogenomic cluster identified is unusual, indicating probable misclassifications of these strains.

*A. faecalis* represents the best-studied species of this genus; three subspecies have been described and validly published: *A. faecalis* subsp. *faecalis*; *A. faecalis* subsp. *parafaecalis* (Schroll et al., 2001); and *A. faecalis* subsp. *phenolicus* (Rehfuss and Urban, 2005). The low ANIb values obtained (<92.3%) and MLSA analysis between *A. faecalis* subsp. *faecalis* (DSM 30030^T^) and *A. faecalis* subsp. *phenolicus* (DSM 16503^T^) indicate that they belong to two different phylogenetic lineages. These results suggest that *A. faecalis* subsp. *phenolicus* should be reclassified as a representative of a different species. Cluster III is represented by *A. aquatilis* QD168 and the strains *A. aquatilis* BU33N (Genome accession: GCA_003076515.1), *A. faecalis* JQ135 (Qiu et al., 2017, 2018; Zhang et al., 2018), *A. faecalis* UBA7629 and *A. faecalis* UBA3227 (Parks et al., 2017). Accordingly, identification of strains QD168 and BU33N by 16S rRNA gene sequence analysis showed >99.9% similarity with *A. aquatilis* LMG 22996^T^ (Durán et al., 2019). Although the type strain of *A. aquatilis* has not yet been sequenced, these data indicate the phylogenomic placement of the *A. aquatilis* species to be in the *Alcaligenes* genomic cluster III. *A. aquatilis* QD168 was isolated from hydrocarbon-polluted sediments from Quintero Bay (Valparaiso Region, Chile). In agreement with the hostile conditions for life found in this crude oil-polluted environment (i.e., high salinity, high levels of persistent organic pollutants, and heavy metals), the genome sequence of *A. aquatilis* QD168 revealed a high number of genetic determinants for the degradation of aromatic compounds, response to oxidative stress, and osmoprotection.

Proteobacteria isolated from polluted sites are known to degrade a broad spectrum of aromatic compounds (Chain et al., 2006; McLeod et al., 2006; Pérez-Pantoja et al., 2008, 2012). Genomic sequence analyses of, so-called, “environmental” bacteria, such as *P. putida* KT2440, *Paraburkholderia xenovorans* LB400 and *C. pinatubonensis* JMP134, have revealed versatile metabolisms and a wide enzymatic repertoire for degradation of aromatic compounds (Jiménez et al., 2002; Chain et al., 2006; Pérez-Pantoja et al., 2008). To our knowledge, this is the first genome mining report of *A. aquatilis*, describing degradative potential for a number of aromatic compounds. The remarkable number of genes encoding catabolic aromatic pathways found in strain QD168 revealed its metabolic potential to overcome oil-polluted environments. Seven central catabolic pathways and sixteen peripheral catabolic pathways/reactions for the degradation of aromatic compounds were identified in the QD168 genome. Central catabolic pathways included the catechol, protocatechuate, 3-(2,3-dihydroxyphenyl)-propionate, phenylacetyl-CoA, homogentisate, gentisate, and 2,5-dihydroxynicotinate. Interestingly, strain QD168 was able to grow on benzoate, 3-hydroxybenzoate, 4-hydroxybenzoate, benzene, cinnamate, 3-hydroxycinnamate, nicotinate, tryptophan, anthranilate, 4-aminobenzoate, benzamide, toluene, and biphenyl as sole carbon sources. In agreement with these results, prediction of benzoate (*benABCD*), cinnamate (*hcaBCFE*), nicotinate (*nicABC*), tryptophan (*kynABU*), anthranilate (*andAaAbAcAd*), and benzene (*dmpKLMNOP*) peripheral catabolic pathways were identified in the QD168 genome. However, bioinformatic analyses did not identify genes for 3-hydroxybenzoate, 3-hydroxycinnamate, benzamide, 4-aminobenzoate, biphenyl, and toluene pathways, which are substrates for QD168 growth. In addition, 18 other catabolic genes (i.e., monooxygenases, hydroxylases, and aromatic ring-hydroxylating dioxygenases) could not be affiliated to a specific catalytic function, although they may play key roles in the degradations of these compounds. Further functional analyses are needed to reveal the catabolic roles of these genes of strain QD168 in the degradation of aromatic compounds. Even though, genes encoding toluene catabolic pathway were not found in the QD168 genome, the presence of *dmp* genes (phenol hydroxylase) likely explains QD168 growth on toluene. In *Arthrobacter* sp. W1 and *Comamonas testosteroni* R5, a phenol hydroxylase, encoded by *dmp* genes, catalyzes the oxidation of benzene and toluene (Teramoto et al., 1999; Ma et al., 2013).

Among the *Alcaligenes* spp. analyzed in this study, five strains of *A. faecalis* (JQ135, BDB4, P156, DSM 30030^T^, and ZD02) possessed the *dmpKLMNOP* gene cluster. Strains QD168, BU33N and JQ135, members of the *A. aquatilis* phylogenomic cluster (cluster III), possess the *dmp* gene cluster. The presence of the *dmpRKLMNOP* gene cluster, encoding a multicomponent phenol hydroxylase, and a DmpR transcriptional regulator in strain QD168, may explain its ability to degrade benzene. Accordingly, the *dmpP* gene that encodes the phenol hydroxylase protein P5, was up-regulated during growth of *A. aquatilis* QD168 on benzene (Figure 5C). Detection of phenol during benzene-degradation, suggests a monooxygenation catalytic step and subsequent oxidation to catechol, as a central intermediate (Figure 5D). In agreement with the proposed catechol *ortho*-cleavage catabolic route, a significant up-regulation of the *catA* gene was observed (Figure 5C). The phenol catabolic pathway via *meta*-cleavage, encoded by the *dmpKMNLOPQBCDEFGHI* gene cluster, has been reported in *Pseudomonas* sp. CF600 (Shingler et al., 1992). The DmpR transcriptional activator of strain CF600 is regulated by benzene, phenol and catechol (Shingler and Moore, 1994). An *ortho*-cleavage pathway for phenol- and benzene-degradation, encoded by the *phc* gene cluster, has been described in *Pseudomonas* sp. M1 (Santos and Sá-Correia, 2007). The *phc* gene cluster is regulated by the transcription factor, PhcR, possessing high similarity to the DmpR transcriptional regulator of strain CF600 (Shingler and Moore, 1994). Similarly, in strain QD168, degradation of benzene probably occurs via an *ortho*-cleavage pathway (Figure 4D).

Although several strains of *Alcaligenes* spp. have been described for their ability to degrade some aromatic compounds, information about the physiological and stress response of *Alcaligenes* spp. in the presence of environmental pollutants is scarce. Oxidative stress during the degradation of aromatic compounds has been reported (Agulló et al., 2007; Ponce et al., 2011; Kim and Park, 2014). In this study, a broad repertoire of genes encoding antioxidant and detoxifying enzymes were identified in *A. aquatilis* strain QD168, suggesting an efficient response to redox imbalance. Genes-encoding scavenging enzymes were highly represented by catalases (two *cat* genes), alkyl hydroperoxide reductases (four *ahp* genes), thioredoxin (six *trx* genes), and peroxiredoxin/peroxidase (six genes). Moreover, five transcriptional regulators involved in oxidative stress response were identified in the QD168 genome. In *E. coli*, the regulation of the *cat, ahp* and *trx* genes during exposure to H_2_O_2_ is mainly mediated by the transcriptional regulator, OxyR (Imlay, 2013). An ortholog of OxyR regulator was identified in the QD168 genome, as well as two orthologs of the SoxR transcriptional regulator. In bacteria, the redox-sensitive transcriptional activator SoxR responds to redox-active compounds, such as PQ (Pomposiello and Demple, 2001). The OhrR organic hydroperoxide resistance repressor was identified in the QD168 genome, which has been described in *Bacillus subtilis* as a repressor involved in the organic peroxide response (Fuangthong et al., 2001). Moreover, the HTH-type transcriptional regulator, PerR, identified in strain QD168, has been further studied in *B. subtilis* as an inducible transcriptional regulator under H_2_O_2_ exposure (Fuangthong et al., 2002). These results suggest a complex regulatory strategy of strain QD168 responding to oxidative stress under environmental pressures. The phylogeny lineage of OxyR revealed three distinctive OxyR clades, wherein OxyR of *A. aquatilis* QD168 formed a singleton. The SoxR1 and SoxR2 phylogenetic analyses revealed that SoxR1 of *A. aquatilis* QD168 formed a singleton ungrouped with other SoxR orthologs. Interestingly, SoxR2 from strain QD168 grouped with *Gammaproteobacteria* orthologous proteins. Within this main phylogenetic clade, two subgroups of SoxR, including those in enteric (*E. coli* K-12, *S. typhi* CT18 and *S. typhimurium* LT2) and non-enteric bacteria (*P. putida* KT2440 and *A. oleovorans* DR1), were clearly distinguished. In enteric bacteria, the oxidized form of the transcription factor, SoxR, induces the expression of the transcription factor, SoxS, activating more than 100 genes. In non-enteric bacteria, a different response mechanism occurs, in which SoxS is not present. SoxR directly activates, not only the expression of genes involved in antioxidant defense, but also up-regulates mono- and dioxygenases-encoding genes (Dietrich and Kiley, 2011; Kim et al., 2017). Accordingly, SoxR2 of *A. aquatilis* QD168 clustered with proteins from non-enteric bacteria and the *soxS* gene is not present in its genome.

In agreement with the broad repertoire of identified stress response genes, an unusual resistance to PQ (0.2 M) and tolerance to H_2_O_2_ (1 mM) was observed in strain QD168. Notably, inhibitory concentrations of H_2_O_2_ and PQ in strain QD168 were significantly higher compared to those previously reported in *E. coli.* Inhibition of *E. coli* growth has been reported to occur at low H_2_O_2_ concentrations (0.03 mM), inactivating key enzymes containing active-site sulfhydryl residues (Seaver and Imlay, 2001). Resistance to H_2_O_2_ and PQ in strain QD168 may correlate with the number of genes-encoding ROS scavenging enzymes, and transcriptional regulators identified for redox balance. Stress-related transcriptional regulators found in strain QD168 probably regulate the oxidative stress response to H_2_O_2_ and PQ.

One of the most important types of marine environmental stress is a change in the external osmotic conditions. Consequently, bacterial cells have developed a broad and sophisticated repertoire to counteract drastic changes in external osmotic conditions (Krämer, 2010). A mechanism to maintain osmotic balance is the production of compatible solutes as betaines (glycine betaine and derivatives), amino acids (proline, glutamate, glutamine, and alanine) and ectoines (ectoine and hydroxyectoine). *A. aquatilis* QD168 possesses four genes encoding ectoine/5-hydroxyectoine synthesis (*ectABCD*), and four genes encoding transports for these solutes (*ehuBCDA*), which may explain its resistance to 10% (w v^-1^) NaCl (Figure 8). *Alcaligenes* spp. tolerance to NaCl has been reported previously (Lu et al., 2017). *A. aquatilis* CCTCC 2015279^T^ showed a salt tolerance to 7% (w v^-1^) NaCl, while *A. faecalis* subsp. *faecalis* CGMCC 11786^T^, *A. faecalis* subsp. *parafaecalis* DSM 13975^T^ and *A. faecalis* subsp. *phenolicus* DSM 16503^T^ tolerate 8% (w v^-1^) NaCl. *A. endophyticus* AER10^T^ showed tolerance to 10% (w v^-1^) NaCl (Lu et al., 2017). The ectoine biosynthetic cluster has been reported in *A. faecalis* subsp. *phenolicus* MB207 (Basharat et al., 2018). The presence of ectoine hydroxylase (*ectD*) gene within the *ect* gene cluster of strain QD168 is probably also involved in its high tolerance to salt stress. The production of 5-hydroxyectoine by ectoine hydroxylase confers additional protective properties (Pastor et al., 2010).

Overall, this study describes a phylogenetic analysis of the marine strain *A. aquatilis* QD168 isolated from a highly polluted environment in Quintero Bay, Central Chile. Genomic and physiological analyses elucidated the adaptive mechanisms of strain QD168 to overcome this hostile environment. *A. aquatilis* QD168 possesses an unusually wide range of enzymatic machinery to survive environmental stressors, including toxic compounds, high salinity, and oxidative stress, which can be suitable for bioremediation processes in unfavorable conditions.

## Author Contributions

RD, VM, BB-S, EC-N, and MS conceived and designed the experiments. RD performed the experiments. RD, VM, BB-S, LR-C, FS-S, EM, EC-N, and MS analyzed the data. MS, EC-N, and BB-S contributed reagents, materials, and analysis tools. RD, VM, BB-S, and LR-C prepared figures and/or tables. RD, VM, BB-S, LR-C, FS-S, EM, and MS authored or reviewed drafts of the manuscript.

## Conflict of Interest Statement

The authors declare that the research was conducted in the absence of any commercial or financial relationships that could be construed as a potential conflict of interest.

## Footnotes

1 http://tree.bio.ed.ac.uk/software/figtree/
