# Supplementary material for: Genomic and Physiological Traits of the Marine Bacterium Alcaligenes aquatilis QD168 Isolated From Quintero Bay, Central Chile, Reveal a Robust Adaptive Response to Environmental Stressors
Source: Front Microbiol. 2019 Apr 5;10:528. doi: 10.3389/fmicb.2019.00528 (PMC6460240; doi:10.3389/fmicb.2019.00528)
Supplement: Supplementary file 1 [file Table_1.docx]

| **Table S1. Genomic dataset *Alcaligenes* spp.** | | | | | | | | | |
| --- | --- | --- | --- | --- | --- | --- | --- | --- | --- |
| **Species** | **Strain** | **Genome state** | **Size (Mb)** | **GC%** | **Contigs** | **Country of isolation** | **Isolation source** | **Accession N°** | **References** |
| ***A. aquatilis*** | **QD168** | **Complete** | **4.32** | **56.4** | **1** | **Chile** | **Crude oil-polluted marine sediment, Quintero Bay, Valparaíso** | **GCA_003671915.1** | **Durán et al. 2019** |
| *A. aquatilis* | BU33N | Chromosome | 3.84 | 56.1 | 4 | Tunisia | Hydrocarbon contaminated marine sediment at a refinery harbor in Bizerte | GCA_003076515.1 | Unpublished |
| *A. faecalis* | BDB4 | Chromosome | 4.24 | 56 | 2 | India | Crude oil-contaminated soil | GCA_002205415.1 | Singha et al. 2017 |
| *A. faecalis* | DSM 30030^T^ | Complete | 4.08 | 56.6 | 1 | - | Human faeces | GCA_002443155.1 | Unpublished |
| *A. faecalis* | JQ135 | Complete | 4.08 | 55.9 | 1 | China | Municipal wastewater | GCA_002242175.1 | Qiu et al. 2017, Qiu et al. 2018 |
| *A. faecalis* | P156 | Complete | 4.05 | 56.7 | 1 | China | Solid waste treatment plant | GCA_001641975.2 | Unpublished |
| *A. faecalis* | ZD02 | Complete | 4.25 | 56.8 | 2 | China | *Caenorhabditis elegans* cadavers | GCA_000967305.2 | Ju et al. 2016 |
| *A. faecalis* | GZAF1 | Contig | 4.39 | 56.8 | 61 | Palestine | Wound human sample, Al-Shifa Hospital | GCA_002120075.1 | Laham et al. 2017 |
| *A. faecalis* | GZAF3 | Contig | 4.35 | 56.8 | 31 | Palestine | Urine human sample, Al-Shifa Hospital | GCA_002119995.1 | Laham et al. 2017 |
| *A. faecalis* | GZAF5 | Contig | 4.44 | 56.9 | 58 | Palestine | Wound human sample, Al-Shifa Hospital | GCA_002120045.1 | Laham et al. 2017 |
| *A. faecalis* | MOR02 | Contig | 4.4 | 56.4 | 23 | Mexico | Hemolymph of a *Galleria mellonella* larvae cadaver | GCA_000770015.1 | Quiroz-Castañeda et al. 2015 |
| *A. faecalis* | NBIB-017 | Scaffold | 4.17 | 56.4 | 17 | China | Rice paddy field in Hubei Province | GCA_001530325.1 | Liu et al. 2016 |
| *A. faecalis* subs. faecalis | NCIB 8687 | Contig | 3.9 | 57.2 | 186 | USA | Soil sample from the courtyard of Rensselaer Polytechnic Institute | GCA_000275465.1 | Unpublished |
| *A. faecalis* subs. faecalis | NCTC 10388 | Contig | 4.25 | 56.3 | 8 | UK | Human wound, Bristol | GCA_900445215.1 | Unpublished |
| *A. faecalis* subs. phenolicus | DSM 16503^T^ | Scaffold | 4.25 | 56.4 | 27 | USA | Continuous graywater bioprocessor, Johnson Space Center, Houston, Texas | GCA_000429385.1 | Rehfuss et al. 2005 |
| *A. faecalis* subs. phenolicus | IITR89 | Scaffold | 3.77 | 57.6 | 23 | India | River Cauvery in Erode, India | GCA_001516865.1 | Regar et al. 2016 |
| *A. faecalis* subs. phenolicus | MB207 | Contig | 4.16 | 56.4 | 9 | Pakistan | Effluent of a tannery in Multan | GCA_002082085.1 | Basharat et al. 2018 |
| *A. faecalis* | UBA11285 | Scaffold | 3.92 | 55.4 | 16 | Australia | Cheese sample, (metagenome) | GCA_003521065.1 | Parks et al. 2017 |
| *A. faecalis* | UBA3227 | Scaffold | 4.05 | 56 | 11 | USA | Metal, New York City MTA subway (metagenome) | GCA_002362965.1 | Parks et al. 2017 |
| *A. faecalis* | UBA3878 | Scaffold | 3.68 | 57.2 | 16 | USA | Wood, New York City MTA subway (metagenome) | GCA_002392125.1 | Parks et al. 2017 |
| *A. faecalis* | UBA7605 | Scaffold | 3.99 | 56.4 | 8 | USA | Wood, New York City MTA subway (metagenome) | GCA_002476455.1 | Parks et al. 2017 |
| *A. faecalis* | UBA7622 | Scaffold | 3.93 | 55.9 | 9 | USA | Wood, New York City MTA subway (metagenome) | GCA_002484125.1 | Parks et al. 2017 |
| *A. faecalis* | UBA7629 | Scaffold | 4.17 | 56.9 | 15 | USA | Metal, New York City MTA subway (metagenome) | GCA_002484005.1 | Parks et al. 2017 |
| *A. faecalis* | YBY | Contig | 4.33 | 56.6 | 11 | China | Activated sludge | GCA_003122065.1 | Unpublished |
| *Alcaligenes* sp. | EGD-AK7 | Contig | 4.28 | 56.6 | 69 | India | Indian agricultural soil | GCA_000465875.3 | Sagarkar et al. 2014 |
| *Alcaligenes* sp. | HPC1271 | Contig | 4.27 | 56.6 | 78 | India | Activated sludge of an effluent treatment plant that treats industrial wastewater | GCA_000313875.1 | Kapley et al. 2013 |

(GenBank, September 2018)
