# Supplementary material for: Genomic and Physiological Traits of the Marine Bacterium Alcaligenes aquatilis QD168 Isolated From Quintero Bay, Central Chile, Reveal a Robust Adaptive Response to Environmental Stressors
Source: Front Microbiol. 2019 Apr 5;10:528. doi: 10.3389/fmicb.2019.00528 (PMC6460240; doi:10.3389/fmicb.2019.00528)
Supplement: Supplementary file 2 [file Table_2.docx]

| **Table S2. Percentages of Clusters of Orthologous Groups Category of *Alcaligenes* spp. Strains.** | | | | | | | | | | |  | |  | |  |
| --- | --- | --- | --- | --- | --- | --- | --- | --- | --- | --- | --- | --- | --- | --- | --- |
| **Tabla** |  |  |  |  |  | |  | |  | |  | |  | |  |
| **Clusters of Orthologous  Groups Category** | ***A. aquatilis* QD168** | ***A. aquatilis* BU33N** | ***A. faecalis* ZD02** | ***A. faecalis* DSM 30030^T^** | | ***A. faecalis* subsp. *phenolicus* DSM 16503^T^** | | ***A. faecalis* JQ135** | | ***A. faecalis* subsp. *phenolicus* MB207** | | ***A. faecalis* GZAF5** | | ***Alcaligenes* sp. EGD-AK7** | |
| **[D]** Cell cycle control. cell division. chromosome partitioning | 0.75 | 0.74 | 0.75 | 0.76 | 0.73 | | 0.77 | | 0.73 | | 0.8 | | 0.72 | |  |
| **[M]** Cell wall/membrane/envelope biogenesis | 4.34 | 4.85 | 4.79 | 4.61 | 4.68 | | 4.69 | | 4.72 | | 4.64 | | 4.71 | |  |
| **[N]** Cell motility | 1.08 | 1.14 | 1.11 | 1.19 | 1.11 | | 1.06 | | 1.13 | | 1.02 | | 1.1 | |  |
| **[O]** Post-translational modification, protein turnover, and chaperones | 3.51 | 3.72 | 3.45 | 3.48 | 3.37 | | 3.51 | | 3.44 | | 3.35 | | 3.36 | |  |
| **[T]** Signal transduction mechanisms | 2.91 | 3.09 | 3.32 | 3.11 | 3.2 | | 2.96 | | 3.44 | | 2.99 | | 2.84 | |  |
| **[U]** Intracellular trafficking, secretion, and vesicular transport | 2.68 | 1.7 | 1.96 | 1.61 | 2.89 | | 2.05 | | 1.73 | | 2.16 | | 2.04 | |  |
| **[V]** Defense mechanisms | 1.03 | 1.02 | 1.18 | 1.13 | 1.16 | | 1.25 | | 1.23 | | 1.21 | | 1.02 | |  |
| **[W]** Extracellular structures | 0.0 | 0.03 | 0.03 | 0.03 | 0.05 | | 0.05 | | 0.05 | | 0.05 | | 0.05 | |  |
| **[A]** RNA processing and modification | 0.03 | 0.03 | 0.03 | 0.03 | 0.03 | | 0.03 | | 0.03 | | 0.02 | | 0.02 | |  |
| **[B]** Chromatin structure and dynamics | 0.08 | 0.09 | 0.08 | 0.08 | 0.08 | | 0.08 | | 0.08 | | 0.07 | | 0.07 | |  |
| **[J]** Translation, ribosomal structure and biogenesis | 4.34 | 4.88 | 4.56 | 4.64 | 4.48 | | 4.61 | | 4.67 | | 4.32 | | 4.31 | |  |
| **[K]**Transcription | 7.95 | 8.09 | 8.63 | 8.22 | 8.16 | | 7.8 | | 8.64 | | 8.84 | | 7.87 | |  |
| **[L]** Replication, recombination and repair | 5.11 | 3.46 | 3.48 | 3.74 | 4.08 | | 3.81 | | 3.28 | | 4.05 | | 3.59 | |  |
| **[C]** Energy production and conversion | 6.19 | 7.18 | 6.72 | 6.85 | 6.42 | | 6.71 | | 6.75 | | 6.39 | | 6.33 | |  |
| **[E]** Amino acid transport and metabolism | 7.4 | 8.37 | 7.96 | 8.36 | 7.75 | | 7.93 | | 8.22 | | 7.62 | | 7.62 | |  |
| **[F]** Nucleotide transport and metabolism | 2.13 | 2.47 | 2.27 | 2.24 | 2.14 | | 2.26 | | 2.34 | | 2.14 | | 2.19 | |  |
| **[G]** Carbohydrate transport and metabolism | 3.91 | 4.34 | 4.46 | 4.24 | 4.0 | | 4.1 | | 4.38 | | 4.27 | | 4.01 | |  |
| **[H]** Coenzyme transport and metabolism | 3.06 | 3.38 | 3.17 | 3.14 | 2.94 | | 3.25 | | 3.1 | | 3.01 | | 2.94 | |  |
| **[I]** Lipid transport and metabolism | 3.01 | 3.26 | 3.24 | 3.32 | 3.4 | | 3.25 | | 3.31 | | 3.08 | | 3.21 | |  |
| **[P]** Inorganic ion transport and metabolism | 6.34 | 6.78 | 7.24 | 6.91 | 6.72 | | 6.68 | | 7.11 | | 6.7 | | 6.28 | |  |
| **[Q]** Secondary metabolites biosynthesis, transport, and catabolism | 3.06 | 3.32 | 3.24 | 3.29 | 3.1 | | 3.14 | | 3.28 | | 3.11 | | 2.94 | |  |
| **[S]** Function unknown | 23.67 | 21.06 | 21.94 | 21.43 | 21.6 | | 21.46 | | 21.23 | | 22.85 | | 23.89 | |  |
| Unclassified | 7.45 | 7.01 | 6.41 | 7.59 | 7.93 | | 8.55 | | 7.11 | | 7.31 | | 8.87 | |  |
