# Supplementary material for: Genomic and Physiological Traits of the Marine Bacterium Alcaligenes aquatilis QD168 Isolated From Quintero Bay, Central Chile, Reveal a Robust Adaptive Response to Environmental Stressors
Source: Front Microbiol. 2019 Apr 5;10:528. doi: 10.3389/fmicb.2019.00528 (PMC6460240; doi:10.3389/fmicb.2019.00528)
Supplement: Supplementary file 3 [file Table_3.docx]

| **Table S3. Central aromatic pathways predicted in *A. aquatilis* QD168** | | | | | | | | | |  |
| --- | --- | --- | --- | --- | --- | --- | --- | --- | --- | --- |
| **Central Pathway** | **Gene** | **ORF** | **CDS** | **Function** | | | **Organism (identity %/similarity %)** | | **Accession N°** | |
| Catechol | *catR* | D3M96_09450 | CatR | Transcriptional regulator, (LysR family) | | | *Acinetobacter iwoffii* K24 (60/79) | | [O33945.1](https://www.ncbi.nlm.nih.gov/protein/5915888?report=genbank&log$=protalign&blast_rank=1&RID=KGS1YBYF014) | |
|  | *catB* | D3M96_09455 | CatB | Muconate cycloisomerase | | | *A. iwoffii* K24 (64/78) | | [O33946.1](https://www.ncbi.nlm.nih.gov/protein/6014740?report=genbank&log$=protalign&blast_rank=1&RID=KGS1YBYF014) | |
|  | *catA* | D3M96_09460 | CatA | Catechol 1,2-dioxygenase | | | *Acinetobacter* sp. ADP1 (73/85) | | [P07773.1](https://www.ncbi.nlm.nih.gov/protein/115695?report=genbank&log$=protalign&blast_rank=1&RID=KGS1YBYF014) | |
|  | *catI* | D3M96_09465 | CatI | 3-Oxoadipate CoA-transferase subunit A | | | *Pseudomonas putida* (71/86) | | [Q01103.2](https://www.ncbi.nlm.nih.gov/protein/1172040?report=genbank&log$=protalign&blast_rank=1&RID=KGS1YBYF014) | |
|  | *catJ* | D3M96_09470 | CatJ | 3-Oxoadipate CoA-transferase subunit B | | | *P. putida* KT2440 (68/81) | | [P0A101.2](https://www.ncbi.nlm.nih.gov/protein/60392887?report=genbank&log$=protalign&blast_rank=1&RID=KGS1YBYF014) | |
|  | *catD* | D3M96_09475 | CatD | 3-Oxoadipate enol-lactonase | | | *Acinetobacter* sp. ADP1 (53/67) | | [P00632.3](https://www.ncbi.nlm.nih.gov/protein/6166146?report=genbank&log$=protalign&blast_rank=1&RID=KGS1YBYF014) | |
|  | *catC* | D3M96_09480 | CatC | Muconolactone delta-isomerase | | | *A. iwoffii* K24 (69/81) | | [O33947.1](https://www.ncbi.nlm.nih.gov/protein/6014743?report=genbank&log$=protalign&blast_rank=1&RID=KGS1YBYF014) | |
| Protocatechuate | *ligC* | D3M96_07050 | LigC | 4-Carboxy-2-hydroxymuconate-6-semialdehyde dehydrogenase | | | *Sphingomonas paucimobilis* SYK6 (73/82) | | [Q9KWL3.1](https://www.ncbi.nlm.nih.gov/protein/75488711?report=genbank&log$=protalign&blast_rank=1&RID=M5FKFFVS014) | |
|  | *ligB* | D3M96_07055 | LigB | Protocatechuate 4,5-dioxygenase beta subunit | | | *S. paucimobilis* SYK6 (58/78) | | [P22636.1](https://www.ncbi.nlm.nih.gov/protein/129717?report=genbank&log$=protalign&blast_rank=1&RID=M5FKFFVS014) | |
|  | *ligA* | D3M96_07060 | LigA | Protocatechuate 4,5-dioxygenase alfa subunit | | | *S. paucimobilis* SYK6 (61/79) | | [P22635.1](https://www.ncbi.nlm.nih.gov/protein/129716?report=genbank&log$=protalign&blast_rank=1&RID=M5FKFFVS014) | |
|  | *ligI* | D3M96_07065 | LigI | 2-Pyrone-4,6-dicarboxylate hydrolase | | | *Comamonas* sp. E6 (75/87) | | [Q93PS7.1](https://www.ncbi.nlm.nih.gov/protein/75408934?report=genbank&log$=protalign&blast_rank=1&RID=M5FKFFVS014) | |
|  | *ligK* | D3M96_07070 | LigK | 4-Carboxy-4-hydroxy-2-oxoadipate aldolase | | | *Pseudomonas straminea* (92/95) | | [Q9AQI0.1](https://www.ncbi.nlm.nih.gov/protein/75411441?report=genbank&log$=protalign&blast_rank=1&RID=M5FKFFVS014) | |
|  | *ligJ* | D3M96_07075 | LigJ | 4-Oxalomesaconate hydrolase | | | *Acidovorax* sp. T1 (94/97) | | WP_08774320 | |
|  | *pcaK* | D3M96_07080 | PcaK | 4-Hydroxybenzoate transporter | | | *P. putida* KT2440 (49/67) | | [Q51955.1](https://www.ncbi.nlm.nih.gov/protein/6093655?report=genbank&log$=protalign&blast_rank=1&RID=M5FKFFVS014) | |
|  | *galD* | D3M96_07085 | GalD | 4-Oxalomesaconate tautomerase | | | *P. putida* KT2440 (49/64) | | [Q88JY0.1](https://www.ncbi.nlm.nih.gov/protein/81441141?report=genbank&log$=protalign&blast_rank=1&RID=M5FKFFVS014) | |
|  | *pcaQ* | D3M96_07090 | LysR | Transcriptional regulator (LysR family) | | | *E. coli K-12* (35/57) | | Q47005.1 | |
| 3-(2,3-Dihydroxyphenyl)-propionate/2,3-dihydroxy-cinnamate | *mhpB* | D3M96_14800 | MhpB | 3-(2,3-Dihydroxyphenyl)-propionate/2,3-dihydroxycinnamate 1,2-dioxygenase | | | *P. putida* (59/74) | | [Q9F9U5.1](https://www.ncbi.nlm.nih.gov/protein/Q9F9U5.1?report=genbank&log$=protalign&blast_rank=1&RID=K9RH5T3M014) | |
|  | *mhpC* | D3M96_14805 | MhpC | 2-Hydroxy-6-oxononadiendioate/2-hydroxy-6-oxononatriendioate hydrolase | | | *Klebsiella pneumoniae* MGH 78578 (76/87) | | [A6TAC7.1](https://www.ncbi.nlm.nih.gov/protein/A6TAC7.1?report=genbank&log$=protalign&blast_rank=1&RID=K9RH5T3M014) | |
|  | *mhpD* | D3M96_14810 | MhpD | 2-Keto-4-pentenoate hydratase | | | *P. putida* (69/81) | | [Q49KF9.1](https://www.ncbi.nlm.nih.gov/protein/Q49KF9.1?report=genbank&log$=protalign&blast_rank=1&RID=K9TNEXCV014) | |
|  | *mhpF* | D3M96_14815 | MhpF | Acetaldehyde dehydrogenase | | | *Cupriavidus necator* H16 (78/88) | | [Q0K3S6.1](https://www.ncbi.nlm.nih.gov/protein/Q0K3S6.1?report=genbank&log$=protalign&blast_rank=1&RID=K9TNEXCV014) | |
|  | *mhpE* | D3M96_14820 | MhpE | 4-Hydroxy-2-oxovalerate aldolase | | | *Dechloromonas aromatica* RCB (90/96) | | [Q47B13.1](https://www.ncbi.nlm.nih.gov/protein/Q47B13.1?report=genbank&log$=protalign&blast_rank=1&RID=K9MED0HP01R) | |
|  | *mhpT* | D3M96_14825 | MhpT | 3-(3-Hydroxyphenyl)-propionate transporter | | | *E. coli* K-12 (44/64) | | [P77589.2](https://www.ncbi.nlm.nih.gov/protein/P77589.2?report=genbank&log$=protalign&blast_rank=1&RID=K9MED0HP01R) | |
| Phenylacetyl-CoA | *paaA* | D3M96_12710 | PaaA | 1,2-Phenylacetyl-CoA epoxidase, subunit A | | | *E. coli* K-12 (68/82) | | [P76077.1](https://www.ncbi.nlm.nih.gov/protein/3025108?report=genbank&log$=protalign&blast_rank=1&RID=KDS0HRAK015) | |
|  | *paaB* | D3M96_12705 | PaaB | 1,2-Phenylacetyl-CoA epoxidase, subunit B | | | *E. coli* K-12 (63/80) | | [P76078.1](https://www.ncbi.nlm.nih.gov/protein/3025248?report=genbank&log$=protalign&blast_rank=1&RID=KDS0HRAK015) | |
|  | *paaC* | D3M96_12700 | PaaC | 1,2-Phenylacetyl-CoA epoxidase, subunit C | | | *E. coli* K-12 (48/63) | | [P76079.1](https://www.ncbi.nlm.nih.gov/protein/3025109?report=genbank&log$=protalign&blast_rank=1&RID=KDS0HRAK015) | |
|  | *paaD* | D3M96_12695 | PaaD | 1,2-Phenylacetyl-CoA epoxidase, subunit D | | | *E. coli* K-12 (49/65) | | [P76080.2](https://www.ncbi.nlm.nih.gov/protein/269849642?report=genbank&log$=protalign&blast_rank=1&RID=KDS0HRAK015) | |
|  | *paaE* | D3M96_12690 | PaaE | 1,2-Phenylacetyl-CoA epoxidase, subunit E | | | *E. coli* K-12 (46/64) | | [P76081.1](https://www.ncbi.nlm.nih.gov/protein/13632702?report=genbank&log$=protalign&blast_rank=1&RID=KDS0HRAK015) | |
|  | *paaZ* | D3M96_12685 | PaaZ | Oxepin-CoA hydrolase | | | *E. coli* K-12 (27/45) | | [P77455.1](https://www.ncbi.nlm.nih.gov/protein/P77455.1?report=genbank&log$=protalign&blast_rank=2&RID=K9AAN74601R) | |
|  | *paaG* | D3M96_12680 | PaaG | 1,2-Epoxyphenylacetyl-CoA isomerase | | | *E. coli* K-12 (58/73) | | [P77467.1](https://www.ncbi.nlm.nih.gov/protein/3334290?report=genbank&log$=protalign&blast_rank=1&RID=KDS0HRAK015) | |
|  | *paaI* | D3M96_12675 | PaaI | Acyl-coenzyme A thioesterase | | | *E. coli* K-12 (53/70) | | P76084.1 | |
| Homogentisate | *hmgC* | D3M96_13400 | HmgC | | Maleylacetoacetate isomerase | | *Pseudomonas aeruginosa* PAO1 (47/64) | | [P57109](http://www.uniprot.org/uniprot/P57109) | |
|  | *hmgA* | D3M96_03780 | HmgA | | Homogentisate 1,2-dioxygenase | | *Bordetella avium* 197N (77/84) | | [Q2KYC8.1](https://www.ncbi.nlm.nih.gov/protein/123514473?report=genbank&log$=protalign&blast_rank=1&RID=MGX33TPT013) | |
|  | *hmgB* | D3M96_03775 | HmgB | | Fumarylacetoacetase | *B. pertussis* Tohama I (65/78) | | Q7VUG1 | |  |
| 2,5-Dihydroxy nicotinate | *nicE* | D3M96_17640 | NicE | | Maleate isomerase | *Alcaligenes faecalis* (98/98) | | O24766.1 | |  |
|  | *nicD* | D3M96_16080 | NicD | | N-formylmaleamate deformylase | *P. putida* KT2440 (59/73) | | [Q88FY3.1](https://www.ncbi.nlm.nih.gov/protein/81586024?report=genbank&log$=protalign&blast_rank=1&RID=MNV7NV2V015) | |  |
|  | *nicX* | D3M96_16075 | NicX | | 2,5-Dihydroxypiridine 5,6-dioxygenase | *P. putida* KT2440 (55/70) | | [Q88FY1.1](https://www.ncbi.nlm.nih.gov/protein/81440183?report=genbank&log$=protalign&blast_rank=1&RID=MNV7NV2V015) | |  |
|  | *nicF* | D3M96_16070 | NicF | | Maleamate amidohydrolase | *P. putida* KT2440 (43/56) | | [Q88FY5.1](https://www.ncbi.nlm.nih.gov/protein/81440186?report=genbank&log$=protalign&blast_rank=1&RID=MNV7NV2V015) | |  |
|  | *nicD2* | D3M96_15900 | NicD2 | | N-formylmaleamate deformylase | *P. putida* KT2440 (60/73) | | [Q88FY3.1](https://www.ncbi.nlm.nih.gov/protein/Q88FY3.1?report=genbank&log$=protalign&blast_rank=1&RID=K9JJPGT0015) | |  |
|  | *nicX2* | D3M96_15895 | NicX2 | | 2,5-dihydroxypiridine 5,6-dioxygenase | *P. putida* KT2440 (55/70) | | [Q88FY1.1](https://www.ncbi.nlm.nih.gov/protein/Q88FY1.1?report=genbank&log$=protalign&blast_rank=1&RID=K9JJPGT0015) | |  |
|  | *nicF2* | D3M96_15890 | NicF2 | | Maleamate amidohydrolase | *P. putida* KT2440 (43/53) | | [Q88FY5.1](https://www.ncbi.nlm.nih.gov/protein/Q88FY5.1?report=genbank&log$=protalign&blast_rank=1&RID=K9JJPGT0015) | |  |
| Gentisate | *sdgD* | D3M96_00260 | SdgD | | Gentisate 1,2-dioxygenase | *Streptomyces* sp. (37/51) | | [Q7X284.1](https://www.ncbi.nlm.nih.gov/protein/75388302?report=genbank&log$=protalign&blast_rank=1&RID=MJCVGEBC013) | |  |
|  | *nagL* | D3M96_13400 | NagL1 | | Maleylpyruvate isomerase | *Vibrio cholerae* N16961 (46/69) | | [Q9KSB2.1](https://www.ncbi.nlm.nih.gov/protein/Q9KSB2.1?report=genbank&log$=protalign&blast_rank=1&RID=KD3DYKAZ014) | |  |
|  | *nagK* | D3M96_11765 | NagK | | Fumarylpyruvate hydrolase | *Ralstonia* sp. (42/64) | | [O86042.1](https://www.ncbi.nlm.nih.gov/protein/O86042.1?report=genbank&log$=protalign&blast_rank=2&RID=KD42TBWT015) | |  |
