# Supplementary material for: Genomic and Physiological Traits of the Marine Bacterium Alcaligenes aquatilis QD168 Isolated From Quintero Bay, Central Chile, Reveal a Robust Adaptive Response to Environmental Stressors
Source: Front Microbiol. 2019 Apr 5;10:528. doi: 10.3389/fmicb.2019.00528 (PMC6460240; doi:10.3389/fmicb.2019.00528)
Supplement: Supplementary file 4 [file Table_4.docx]

| **Table S4. Peripheral aromatic pathways predicted in *A. aquatilis* QD168** | | | | | | | | |
| --- | --- | --- | --- | --- | --- | --- | --- | --- |
| **Central pathway** | **Peripheral  pathway** | **Gene** | **ORF** | **CDS** | **Function** | **Organism (identity %/similarity %)** | **Accession N°** |  |
| Catechol | Benzoate | *benE* | D3M96_09800 | BenE | Benzoate transporter | *Acinetobacter.* sp. ADP1 (46/68) | [O30513.2](https://www.ncbi.nlm.nih.gov/protein/51704234?report=genbank&log$=protalign&blast_rank=1&RID=KETWRXYJ015) |  |
|  |  | *benD* | D3M96_09795 | BenD | 1,2-dihydroxy-3,4-cyclohexadiene-1-carboxylate dehydrogenase | *P. putida* (66/76) | [P23102.1](https://www.ncbi.nlm.nih.gov/protein/139853?report=genbank&log$=protalign&blast_rank=1&RID=KETWRXYJ015) |  |
|  |  | *benC* | D3M96_09790 | BenC | Benzoate 1,2-dioxygenase oxidoreductase | *Acinetobacter.* sp. ADP1 (51/67) | [P07771.2](https://www.ncbi.nlm.nih.gov/protein/51704250?report=genbank&log$=protalign&blast_rank=2&RID=KETWRXYJ015) |  |
|  |  | *benB* | D3M96_09785 | BenB | Benzoate 1,2-dioxygenase beta subunit | *Burkholderia cepacia* 2CBS (58/73) | [P07770.1](https://www.ncbi.nlm.nih.gov/protein/114916?report=genbank&log$=protalign&blast_rank=3&RID=KETWRXYJ015) |  |
|  |  | *benA* | D3M96_09780 | BenA | Benzoate 1,2-dioxygenase alpha subunit | *B. cepacia* 2CBS (63/77) | [P07769.2](https://www.ncbi.nlm.nih.gov/protein/21431747?report=genbank&log$=protalign&blast_rank=2&RID=KETWRXYJ015) |  |
|  |  | *benM* | D3M96_09775 | BenM | Transcriptional regulator, LysR family | *Acinetobacter. sp. ADP1* (41/64) | [O68014.2](https://www.ncbi.nlm.nih.gov/protein/51704241?report=genbank&log$=prottop&blast_rank=2&RID=KETWRXYJ015) |  |
|  | Phenol/Benzene | *dmpP* | D3M96_09760 | DmpP | Phenol hydroxylase protein P5 | *Acinetobacter pittii* PHEA-2 (73/84) | [Q7WTJ2.3](https://www.ncbi.nlm.nih.gov/protein/39931048?report=genbank&log$=protalign&blast_rank=1&RID=KETWRXYJ015) |  |
|  |  | *dmpO* | D3M96_09755 | DmpO | Phenol hydroxylase protein P4 | *Pseudomonas* sp. CF600 (40/61) | [P19733.1](https://www.ncbi.nlm.nih.gov/protein/118695?report=genbank&log$=protalign&blast_rank=1&RID=KETWRXYJ015) |  |
|  |  | *dmpN* | D3M96_09750 | DmpN | Phenol hydroxylase protein P3 | *Pseudomonas* sp. CF600 (72/83) | [P19732.1](https://www.ncbi.nlm.nih.gov/protein/118694?report=genbank&log$=protalign&blast_rank=1&RID=KETWRXYJ015) |  |
|  |  | *dmpM* | D3M96_09745 | DmpM | Phenol hydroxylase protein P2 | *Pseudomonas* sp. CF600 (59/76) | [P19731.1](https://www.ncbi.nlm.nih.gov/protein/118693?report=genbank&log$=protalign&blast_rank=1&RID=KETWRXYJ015) |  |
|  |  | *dmpL* | D3M96_09740 | DmpL | Phenol hydroxylase protein P1 | *A. pittii* PHEA-2 (60/75) | [Q7WTJ6.3](https://www.ncbi.nlm.nih.gov/protein/39931049?report=genbank&log$=protalign&blast_rank=1&RID=KETWRXYJ015) |  |
|  |  | *dmpK* | D3M96_09735 | DmpK | Phenol hydroxylase protein P0 | *Pseudomonas* sp. CF600 (53/66) | [P19729.1](https://www.ncbi.nlm.nih.gov/protein/118691?report=genbank&log$=protalign&blast_rank=1&RID=KETWRXYJ015) |  |
|  |  | *dmpR* | D3M96_09730 | DmpR | Transcriptional regulator (Phenol) | *Acinetobacter guillouiae* NCIB 8250 (56/72) | [Q43965.1](https://www.ncbi.nlm.nih.gov/protein/75346195?report=genbank&log$=protalign&blast_rank=1&RID=KETWRXYJ015) |  |
|  | Anthranilate | *andAd* | D3M96_16060 | AndAd | Anthranilate 1,2-dioxygenase small subunit | *B. cepacia* (35/57) | [Q84BZ2.1](https://www.ncbi.nlm.nih.gov/protein/Q84BZ2.1?report=genbank&log$=protalign&blast_rank=1&RID=K9FXXYH4015) |  |
|  |  | *andAc* | D3M96_16055 | AndAc | Anthranilate 1,2-dioxygenase large subunit | *B. cepacia* (42/63) | [Q84BZ3.1](https://www.ncbi.nlm.nih.gov/protein/Q84BZ3.1?report=genbank&log$=protalign&blast_rank=2&RID=K9FXXYH4015) |  |
|  |  | *andAb* | D3M96_16050 | AndAb | Anthranilate 1,2-dioxygenase reductase component | *B. cepacia* (33/49) | [Q84BZ0.1](https://www.ncbi.nlm.nih.gov/protein/Q84BZ0.1?report=genbank&log$=protalign&blast_rank=9&RID=K9FXXYH4015) |  |
|  |  | *andAa* | D3M96_16045 | AndAa | Anthranilate 1,2-dioxygenase ferredoxin subunit | *B. cepacia* (58/72) | [Q84BZ1.1](https://www.ncbi.nlm.nih.gov/protein/Q84BZ1.1?report=genbank&log$=protalign&blast_rank=8&RID=K9FXXYH4015) |  |
|  | Tryptophan | *kynA* | D3M96_18575 | KynA | Tryptophan 2,3-dioxygenase | *Polaromonas naphthalenivorans* CJ2 (81/88) | [A1VRP1.1](https://www.ncbi.nlm.nih.gov/protein/A1VRP1.1?report=genbank&log$=protalign&blast_rank=1&RID=K9HAS7EP014) |  |
|  |  | *kynB* | D3M96_00275 | KynB | Kynurenine formamidase | *Salinibacter ruber* DSM 13855 (25/37) | [Q2S2F5.1](https://www.ncbi.nlm.nih.gov/protein/Q2S2F5.1?report=genbank&log$=protalign&blast_rank=1&RID=K9HAS7EP014) |  |
|  |  | *kynU1* | D3M96_08330 | KynU1 | Kynureninase | *Deinococcus radiodurans* R1 (31/47) | [Q9RYH5.2](https://www.ncbi.nlm.nih.gov/protein/Q9RYH5.2?report=genbank&log$=protalign&blast_rank=1&RID=K9HAS7EP014) |  |
|  |  | *kynU2* | D3M96_02255 | KynU2 | Kynureninase | *Pseudomonas fluorescens* (57/71) | [P83788.1](https://www.ncbi.nlm.nih.gov/protein/P83788.1?report=genbank&log$=protalign&blast_rank=1&RID=K9HAS7EP014) |  |
| Protocatechuate | 4-Hydroxy benzoate | *phbA* | D3M96_07120 | PhbA | 4-Hydroxybenzoate hydroxylase | *P. aeruginosa* PAO1 (65/76) | [P20586.1](https://www.ncbi.nlm.nih.gov/protein/130074?report=genbank&log$=protalign&blast_rank=1&RID=M5FKFFVS014) |  |
|  | 4-cresol | *pchC* | D3M96_07105 | PchC | 4-cresol dehydrogenase subunit cytochrome c | *P. putida* NCIMB 9869 (44/63) | [P09787.2](https://www.ncbi.nlm.nih.gov/protein/17380392?report=genbank&log$=protalign&blast_rank=1&RID=M5FKFFVS014) |  |
|  |  | *pchF* | D3M96_07115 | PchF | 4-cresol dehydrogenase subunit flavoprotein | *P. putida* NCIMB 9869 (77/88) | [P09788.3](https://www.ncbi.nlm.nih.gov/protein/17380393?report=genbank&log$=protalign&blast_rank=1&RID=M5FKFFVS014) |  |
|  |  | *pchA* | D3M96_07100 | PchA | 4-hydroxybenzaldehyde dehydrogenase | *P. putida* NCIMB 9869 (37/57) | [Q59702.2](https://www.ncbi.nlm.nih.gov/protein/75492062?report=genbank&log$=protalign&blast_rank=5&RID=M5FKFFVS014) |  |
|  | 4-carboxy- diphenyl ether | *pobA* | D3M96_00065 | PobA | Phenoxybenzoate dioxygenase alpha subunit | *Pseudomonas pseudoalcaligenes* POB310 (30/46) | [Q52185.1](https://www.ncbi.nlm.nih.gov/protein/3024427?report=genbank&log$=protalign&blast_rank=1&RID=MM4736WN016) |  |
|  |  | *pobB* | D3M96_00060 | PobB | Phenoxybenzoate dioxygenase beta subunit | *P. pseudoalcaligenes* POB310 (40/54) | [Q52186.1](https://www.ncbi.nlm.nih.gov/protein/3024428?report=genbank&log$=protalign&blast_rank=7&RID=MM4736WN016) |  |
| 3-(2,3-Dihydroxyphenyl)-propionate /2,3-dihydroxy-cinnamate | 3-Phenyl-propionate/ cinnamate | *hcaD* | D3M96_14795 | HcaD | 3-Phenylpropionate/cinnamate dioxygenase ferredoxin | *Photorharbus luminicens* subsp. *laumondii* TTO1 (45/63) | [Q7N4V5.1](https://www.ncbi.nlm.nih.gov/protein/Q7N4V5.1?report=genbank&log$=protalign&blast_rank=1&RID=K9RH5T3M014) |  |
|  |  | *hcaB* | D3M96_14790 | HcaB | 3-Phenylpropionate-dihidrodiol/cinnamate-dihidrodiol dehydrogenase | *E. coli* IAI1 (70/80) | [B7M7P4.1](https://www.ncbi.nlm.nih.gov/protein/B7M7P4.1?report=genbank&log$=protalign&blast_rank=1&RID=K9RH5T3M014) |  |
|  |  | *hcaC* | D3M96_14785 | HcaC | 3-Phenylpropionate/cinnamate dioxygenase ferredoxin | *E. coli* O157:H7 (66/76) | [P0ABW1.1](https://www.ncbi.nlm.nih.gov/protein/P0ABW1.1?report=genbank&log$=protalign&blast_rank=2&RID=K9RH5T3M014) |  |
|  |  | *hcaF* | D3M96_14780 | HcaF | 3-Phenylpropionate/cinnamate dioxygenase beta subunit | *P. luminicens* subsp. *laumondii* TTO1 (66/79) | [Q7N4V9.1](https://www.ncbi.nlm.nih.gov/protein/Q7N4V9.1?report=genbank&log$=protalign&blast_rank=2&RID=K9RH5T3M014) |  |
|  |  | *hcaE* | D3M96_14775 | HcaE | 3-phenylpropionate/cinnamate dioxygenase alpha subunit | *P. luminicens* subsp. *laumondii* TTO1 (78/87) | [Q7N4W0.1](https://www.ncbi.nlm.nih.gov/protein/Q7N4W0.1?report=genbank&log$=protalign&blast_rank=6&RID=K9RH5T3M014) |  |
|  |  | *hcaR* | D3M96_14770 | HcaR | Transcripcional activator HcaR | *E. coli* K-12 (46/59) | [Q47141.2](https://www.ncbi.nlm.nih.gov/protein/Q47141.2?report=genbank&log$=protalign&blast_rank=1&RID=K9RH5T3M014) |  |
| Phenylacetyl-CoA | Phenylacetate | *paaK* | D3M96_12670 | PaaK | Phenylacetate-CoA ligase | *Azoarcus evansii* KB 740 (71/84) | [Q9L9C1.1](https://www.ncbi.nlm.nih.gov/protein/75417222?report=genbank&log$=protalign&blast_rank=1&RID=KDS0HRAK015) |  |
|  | Styrene | *styB* | D3M96_00350 | StyB | Flavin reductase NADH-dependent | *P. fluorescens* (36/55) | [O06835.1](https://www.ncbi.nlm.nih.gov/protein/75340319?report=genbank&log$=protalign&blast_rank=7&RID=MJCVGEBC013) |  |
|  |  | *styA* | D3M96_00345 | StyA | Styrene monooxygenase | *P. fluorescens* (30/47) | [O06834.1](https://www.ncbi.nlm.nih.gov/protein/75340318?report=genbank&log$=protalign&blast_rank=1&RID=MJCVGEBC013) |  |
| Homogentisate | 4-Hydroxyphenyl pyruvate | *hpd* | D3M96_15170 | Hpd | 4-Hydroxyphenylpyruvate dioxygenase | *Pseudomonas* sp. P.J. 874 (85/90) | [P80064.1](https://www.ncbi.nlm.nih.gov/protein/P80064.1?report=genbank&log$=protalign&blast_rank=1&RID=K9MED0HP01R) |  |
|  | Phenylpyruvate | *hpd* | D3M96_15170 | Hpd | 4-hydroxyphenylpyruvate dioxygenase | *Pseudomonas* sp. P.J. 874 (85/90) | [P80064.1](https://www.ncbi.nlm.nih.gov/protein/P80064.1?report=genbank&log$=protalign&blast_rank=1&RID=K9MED0HP01R) |  |
|  | Tyrosine | *tyrB1* | D3M96_08960 | TyrB | Aromatic aminoacid aminotransferase | *P. aeruginosa* PAO1 (67/80) | [P72173.2](https://www.ncbi.nlm.nih.gov/protein/P72173.2?report=genbank&log$=protalign&blast_rank=1&RID=K9SG9BX6014) |  |
|  |  | *tyrB2* | D3M96_19035 | TyrB | Aromatic aminoacid aminotransferase | *E. coli* K-12 (55/71) | [P04693.1](https://www.ncbi.nlm.nih.gov/protein/P04693.1?report=genbank&log$=protalign&blast_rank=1&RID=K9SG9BX6014) |  |
| 2,5-Dihydroxy- nicotinate | Nicotinate | *nicT* | D3M96_09560 | NicT | Transport protein NicT | *P. putida* KT2440 (77/87) | [Q88FY6.1](https://www.ncbi.nlm.nih.gov/protein/Q88FY6.1?report=genbank&log$=protalign&blast_rank=1&RID=K9KJ35FA015) |  |
|  |  | *nicC* | D3M96_09555 | NicC | 6-Hydroxynicotinate 3-monooxygenase | *P. putida* KT2440 (84/91) | [Q88FY2.1](https://www.ncbi.nlm.nih.gov/protein/Q88FY2.1?report=genbank&log$=protalign&blast_rank=1&RID=K9KJ35FA015) |  |
|  |  | *nicR* | D3M96_09550 | NicR | Transcriptional repressor type HTH, NicR | *P. putida* KT2440 (71/82) | [Q88FY0.1](https://www.ncbi.nlm.nih.gov/protein/Q88FY0.1?report=genbank&log$=protalign&blast_rank=1&RID=K9KJ35FA015) |  |
|  |  | *nicB1* | D3M96_15915 | NicB1 | Nicotinate dehydrogenase subunit B | *P. putida* KT2440 (41/56) | [Q88FX8.1](https://www.ncbi.nlm.nih.gov/protein/Q88FX8.1?report=genbank&log$=protalign&blast_rank=1&RID=K9JJPGT0015) |  |
|  |  | *nicA* | D3M96_15910 | NicA | Nicotinate dehydrogenase subunit A | *P. putida* KT2440 (63/80) | [Q88FX9.1](https://www.ncbi.nlm.nih.gov/protein/Q88FX9.1?report=genbank&log$=protalign&blast_rank=1&RID=K9JJPGT0015) |  |
|  |  | *nicB2* | D3M96_15905 | NicB2 | Nicotinate dehydrogenase subunit B | *P. putida* KT2440 (58/70) | [Q88FX8.1](https://www.ncbi.nlm.nih.gov/protein/Q88FX8.1?report=genbank&log$=protalign&blast_rank=1&RID=K9JJPGT0015) |  |
| Gentisate | Salicylyl-CoA | *sdgC* | D3M96_00255 | SdgC | Salicylyl-CoA 5-hydroxylase | *Streptomyces* sp. WA46 (40/54) | [Q7X281.1](https://www.ncbi.nlm.nih.gov/protein/75444581?report=genbank&log$=protalign&blast_rank=1&RID=MJCVGEBC013) |  |
