# Supplementary material for: Genomic and Physiological Traits of the Marine Bacterium Alcaligenes aquatilis QD168 Isolated From Quintero Bay, Central Chile, Reveal a Robust Adaptive Response to Environmental Stressors
Source: Front Microbiol. 2019 Apr 5;10:528. doi: 10.3389/fmicb.2019.00528 (PMC6460240; doi:10.3389/fmicb.2019.00528)
Supplement: Supplementary file 5 [file Table_5.docx]

| **Table S5. Genes involved in oxidative stress response of *A. aquatilis* QD168** | | | | | | | |
| --- | --- | --- | --- | --- | --- | --- | --- |
| **Category** | **Gene** | **ORF** | **CDS** | **Function** | **Organism (identity %/similarity %)** | **Accession N°** |  |
| Scavenging enzymes | *ahpC1* | D3M96_12750 | AhpC1 | Alkyl hydroperoxide reductase subunit C (peroxidase) | *Mycobacterium tuberculosis* CDC 1551 (36/55) | [P9WQB6.1](https://www.ncbi.nlm.nih.gov/protein/613779552?report=genbank&log$=protalign&blast_rank=9&RID=JRATS6GX015) |  |
|  | *ahpC2* | D3M96_15655 | AhpC2 | Alkyl hydroperoxide reductase subunit C (peroxidase) | *Salmonella enterica* subsp. enterica serovar Typhimurium LT2 (70/83) | [P0A251.2](https://www.ncbi.nlm.nih.gov/protein/61218500?report=genbank&log$=protalign&blast_rank=2&RID=JRATS6GX015) |  |
|  | *ahpD* | D3M96_11630 | AhpD | Alkyl hydroperoxide reductase AhpD (reductase) | *Bradyrhizobium diazoefficiens* USDA 110 (32/53) | [Q9ANL0.1](https://www.ncbi.nlm.nih.gov/protein/81782972?report=genbank&log$=protalign&blast_rank=3&RID=JRATS6GX015) |  |
|  | *ahpF* | D3M96_15650 | AhpF | Alkyl hydroperoxide reductase subunit AhpF (reductase) | *P. putida* KT2440 (70/82) | [P0A155.1](https://www.ncbi.nlm.nih.gov/protein/60391220?report=genbank&log$=protalign&blast_rank=1&RID=JRATS6GX015) |  |
|  | *bcp* | D3M96_06785 | Bcp | Peroxiredoxin Bcp | *Coxiella burnetii* RSA 493 (48/67) | [Q83CY8.1](https://www.ncbi.nlm.nih.gov/protein/81839069?report=genbank&log$=protalign&blast_rank=1&RID=JRATS6GX015) |  |
|  | *ccp1* | D3M96_07750 | Ccp1 | Cytochrome c551 peroxidase | *Nitrosomonas europaea* ATCC 19718 (34/51) | [P55929.2](https://www.ncbi.nlm.nih.gov/protein/30923250?report=genbank&log$=protalign&blast_rank=4&RID=JRATS6GX015) |  |
|  | *ccp2* | D3M96_04015 | Ccp2 | Cytochrome c551 peroxidase | *N. europaea* ATCC 19718 (65/78) | [P55929.2](https://www.ncbi.nlm.nih.gov/protein/30923250?report=genbank&log$=protalign&blast_rank=1&RID=JRATS6GX015) |  |
|  | *grxC* | D3M96_17565 | GrxC | Glutaredoxin 3 | *P. aeruginosa* PAO1 (52/70) | [Q9HU55.1](https://www.ncbi.nlm.nih.gov/protein/13878512?report=genbank&log$=protalign&blast_rank=1&RID=JRATS6GX015) |  |
|  | *grxD* | D3M96_17835 | GrxD | Glutaredoxin 4 | *Synechocystis* sp. PCC 6803 (57/74) | [P73056.1](https://www.ncbi.nlm.nih.gov/protein/3025188?report=genbank&log$=protalign&blast_rank=1&RID=JRATS6GX015) |  |
|  | *hyPrx5* | D3M96_10550 | HyPrx5 | Peroxiredoxin hybrid hyPrx5 | *Haemophilus influenzae* Rd KW20 (44/57) | [P44758.1](https://www.ncbi.nlm.nih.gov/protein/1723174?report=genbank&log$=protalign&blast_rank=1&RID=JRATS6GX015) |  |
|  | *katA* | D3M96_19795 | KatA | Catalase | *Bordetella pertussis* Tohama I (76/88) | [P0A323.1](https://www.ncbi.nlm.nih.gov/protein/61220322?report=genbank&log$=protalign&blast_rank=1&RID=JRATS6GX015) |  |
|  | *katE* | D3M96_12900 | KatE | Catalase C | *Sinorhizobium meliloti* 1021 (60/73) | [Q9X576.2](https://www.ncbi.nlm.nih.gov/protein/17380489?report=genbank&log$=protalign&blast_rank=1&RID=JR8J6FZ0014) |  |
|  | *prx* | D3M96_04155 | Prx | Peroxiredoxin | *Synechocystis* sp. PCC 6803 substr. Kazusa (42/61) | [P73728.1](https://www.ncbi.nlm.nih.gov/protein/3915480?report=genbank&log$=protalign&blast_rank=1&RID=JRATS6GX015) |  |
|  | *rubA2* | D3M96_16850 | RubA2 | Rubredoxin-2 | *P. aeruginosa* PAO1 (65/79) | [Q9HTK8.1](https://www.ncbi.nlm.nih.gov/protein/81783516?report=genbank&log$=protalign&blast_rank=1&RID=JRATS6GX015) |  |
|  | *sodB1* | D3M96_09520 | SodB1 | Superoxide dismutase [Mn/Fe] | *Methylomonas* sp. J (73/82) | [P23744.1](https://www.ncbi.nlm.nih.gov/protein/134640?report=genbank&log$=protalign&blast_rank=2&RID=JRATS6GX015) |  |
|  | *sodB2* | D3M96_08650 | SodB2 | Superoxide dismutase [Fe] | *B. pertussis* Tohama I (85/89) | [P37369.2](https://www.ncbi.nlm.nih.gov/protein/34978393?report=genbank&log$=protalign&blast_rank=1&RID=JRATS6GX015) |  |
|  | *sodC1* | D3M96_17950 | SodC1 | Superoxide dismutase [Cu-Zn] 1 | *S. enterica* subsp. enterica serovar Typhimurium ST4/74 (60/74) | [E8XDJ8.1](https://www.ncbi.nlm.nih.gov/protein/334351088?report=genbank&log$=protalign&blast_rank=1&RID=JRATS6GX015) |  |
|  | *tlpA* | D3M96_11580 | TlpA | Thioredoxin-like protein TlpA | *B. diazoefficiens* USDA 110 (35/52) | [P43221.1](https://www.ncbi.nlm.nih.gov/protein/1174717?report=genbank&log$=protalign&blast_rank=1&RID=JRATS6GX015) |  |
|  | *trxA* | D3M96_12535 | TrxA | Thioredoxin 1 | *P. aeruginosa* PAO1 (74/87) | [Q9X2T1.1](https://www.ncbi.nlm.nih.gov/protein/13878813?report=genbank&log$=protalign&blast_rank=1&RID=JRATS6GX015) |  |
|  | *trxB* | D3M96_14510 | TrxB | Thioredoxin reductase | *V. cholerae* O1 biovar El Tor N16961 (70/82) | [Q9KSS4.1](https://www.ncbi.nlm.nih.gov/protein/20140856?report=genbank&log$=protalign&blast_rank=1&RID=JRATS6GX015) |  |
|  | *trxC1* | D3M96_11430 | TrxC1 | Thioredoxin 2 | *Streptomyces coelicolor* A3(2) (51/68) | [9RD25.1](https://www.ncbi.nlm.nih.gov/protein/81858668?report=genbank&log$=protalign&blast_rank=1&RID=JRATS6GX015) |  |
|  | *trxC2* | D3M96_15235 | TrxC2 | Thioredoxin 2 | *S. coelicolor* A3(2) (41/48) | [Q9RD25.1](https://www.ncbi.nlm.nih.gov/protein/81858668?report=genbank&log$=protalign&blast_rank=1&RID=JRATS6GX015) |  |
| Transcriptional regulator | *ohrR* | D3M96_17665 | OhrR | Transcriptional regulator of organics peroxide resistance | *B. subtilis* subsp. subtilis 168 (29/49) | [O34777.1](https://www.ncbi.nlm.nih.gov/protein/22095931?report=genbank&log$=protalign&blast_rank=2&RID=JRATS6GX015) |  |
|  | *oxyR* | D3M96_10585 | OxyR | Hydrogen peroxide-inducible genes activator OxyR | *Dickeya chrysanthemi* (42/60) | [P71318.1](https://www.ncbi.nlm.nih.gov/protein/8134603?report=genbank&log$=protalign&blast_rank=1&RID=JRATS6GX015) |  |
|  | *perR* | D3M96_00075 | PerR | HTH-type transcriptional regulator PerR | *E. coli* K-12 (37/51) | [Q57083.1](https://www.ncbi.nlm.nih.gov/protein/2495392?report=genbank&log$=protalign&blast_rank=2&RID=JRATS6GX015) |  |
|  | *soxR1* | D3M96_08910 | SoxR1 | Redox-sensitive transcriptional activator SoxR | *S. enterica* subsp. enterica serovar Typhimurium LT (61/74) | [P0A2R0.1](https://www.ncbi.nlm.nih.gov/protein/60415920?report=genbank&log$=protalign&blast_rank=2&RID=JRATS6GX015) |  |
|  | *soxR2* | D3M96_14980 | SoxR2 | Redox-sensitive transcriptional activator SoxR | *E. coli* O157:H7 (63/76) | [P0ACS3.1](https://www.ncbi.nlm.nih.gov/protein/82583697?report=genbank&log$=protalign&blast_rank=1&RID=JRATS6GX015) |  |
| ROS-resistant isoform | *acnA1* | D3M96_08240 | AcnA1 | Aconitate hydratase A | *Thermus thermophilus* HB8 (50/68) | [Q5SMF6.1](http://www.uniprot.org/uniprot/Q5SMF6) |  |
|  | *acnA2* | D3M96_18610 | AcnA2 | Aconitate hydratase A | *Mycobacterium smegmatis* MC2 155 (68/79) | [A0QX20.1](https://www.ncbi.nlm.nih.gov/protein/302595583?report=genbank&log$=protalign&blast_rank=1&RID=JRATS6GX015) |  |
|  | *fumC* | D3M96_10680 | FumC | Fumarate hydratase, class II | *D. radiodurans* R1 (74/83) | [Q9RR70.1](https://www.ncbi.nlm.nih.gov/protein/39931639?report=genbank&log$=protalign&blast_rank=1&RID=JRATS6GX015) |  |
