# Supplementary material for: Genomic and Physiological Traits of the Marine Bacterium Alcaligenes aquatilis QD168 Isolated From Quintero Bay, Central Chile, Reveal a Robust Adaptive Response to Environmental Stressors
Source: Front Microbiol. 2019 Apr 5;10:528. doi: 10.3389/fmicb.2019.00528 (PMC6460240; doi:10.3389/fmicb.2019.00528)
Supplement: Supplementary file 6 [file Table_6.docx]

| **Table S6. Osmolyte transport and biosynthesis gene prediction in *A. aquatilis* QD168** | | | | | | | | | |
| --- | --- | --- | --- | --- | --- | --- | --- | --- | --- |
| **Osmolyte** | **Process** | **Gene** | **ORF** | **CDS** | **Function** | **Organism (identity %/similarity %)** | **Accesion N°** | |  |
| Ectoine/ 5-hydroxyectoine | Transport | *ehuB* | D3M96_13175 | EhuB | Ectoine/hydroxyectoine ABC transporter substrate-binding protein EhuB | *Nocardiopsis gilva* YIM 90087 (36/54) | | [ASU82347.1.1](https://www.ncbi.nlm.nih.gov/protein/WP_027991260.1?report=genbank&log$=protalign&blast_rank=2&RID=XJHBP0CN014) |  |
|  |  | *ehuC* | D3M96_13180 | EhuC | Ectoine/hydroxyectoine ABC transporter permease subunit EhuC | *S. meliloti* 1021 (49/69) | | [Q92WC7](https://www.uniprot.org/uniprot/Q92WC7) |  |
|  |  | *ehuD* | D3M96_13185 | EhuD | Ectoine/hydroxyectoine ABC transporter permease subunit EhuD | *Sinorhizobium medicae* WSM419 (44/69) | | [A6UFT0](https://www.uniprot.org/uniprot/A6UFT0) |  |
|  |  | *ehuA* | D3M96_13190 | EhuA | Ectoine/hydroxyectoine ABC transporter ATP-binding protein EhuA | *S. medicae* WSM419 (59/75) | | [A6UFT3](https://www.uniprot.org/uniprot/A6UFT3) |  |
|  | Biosynthesis | *ectA* | D3M96_13270 | EctA | Diaminobutyrate acetyltransferase | *Bordetella bronchiseptica* RB50 (57/69) | | Q7WHI7.1 |  |
|  |  | *ectB* | D3M96_13265 | EctB | Diaminobutyrate-2-oxoglutarate transaminase | *B. bronchiseptica* RB50 (78/88) | | [Q7WHI8.1](https://www.ncbi.nlm.nih.gov/protein/Q7WHI8.1?report=genbank&log$=protalign&blast_rank=1&RID=X4WV289D014) |  |
|  |  | *ectC* | D3M96_13260 | EctC | Ectoine synthase | *Bordetella petrii* DSM 12804 (84/91) | | [A9IJZ8.1](https://www.ncbi.nlm.nih.gov/protein/A9IJZ8.1?report=genbank&log$=protalign&blast_rank=1&RID=X4WV289D014) |  |
|  |  | *ectD* | D3M96_13255 | EctD | Ectoine hydroxylase | *B. bronchiseptica* RB50 (69/80) | | [Q7WHJ0.1](https://www.ncbi.nlm.nih.gov/protein/Q7WHJ0.1?report=genbank&log$=protalign&blast_rank=1&RID=X4WV289D014) |  |
| Glutamate | Transport | *gltT1* | D3M96_10385 | GltT1 | Proton/sodium-glutamate symport protein | *Bacillus subtilis* subsp. *subtilis* 168 (37/57) | | [O07605.1](https://www.ncbi.nlm.nih.gov/protein/O07605.1?report=genbank&log$=protalign&blast_rank=3&RID=XGXGH267014) |  |
|  |  | *glnT2* | D3M96_09710 | GltT2 | Glutamate/aspartate import ATP-binding protein | *E. coli O157:H7 (70/81)* | | [P0AAG4.1](https://www.ncbi.nlm.nih.gov/protein/P0AAG4.1?report=genbank&log$=protalign&blast_rank=1&RID=XGXGH267014) |  |
|  |  | *gltS* | D3M96_17165 | GltS | Sodium/glutamate symporter | *H. influenzae* KW20 (37/58) | | [P45240.1](https://www.ncbi.nlm.nih.gov/protein/P45240.1?report=genbank&log$=protalign&blast_rank=1&RID=XGXGH267014) |  |
|  |  | *gadC* | D3M96_12140 | GadC | Glutamate:gamma-aminobutyrate antiporter | *E. coli* K12 (72/86) | | [P63235.1](https://www.ncbi.nlm.nih.gov/protein/P63235.1?report=genbank&log$=protalign&blast_rank=1&RID=XGXGH267014) |  |
|  | Biosynthesis | *gltD* | D3M96_01625 | GltD | Glutamate synthase subunit beta | *M. tuberculosis* CDC1551 (52/66) | | [P9WN18.1](https://www.ncbi.nlm.nih.gov/protein/P9WN18.1?report=genbank&log$=protalign&blast_rank=1&RID=XGXGH267014) |  |
|  |  | *gltB* | D3M96_01630 | GltB | Glutamate synthase subunit alpha | *M. tuberculosis* H37Rv (51/66) | | [P96218.3](https://www.ncbi.nlm.nih.gov/protein/P96218.3?report=genbank&log$=protalign&blast_rank=2&RID=XGXGH267014) |  |
|  |  | *gdhA1* | D3M96_00930 | GdhA1 | Glutamate dehydrogenase | *Thermotoga maritima* MSB8 (56/71) | | [P96110.4](https://www.ncbi.nlm.nih.gov/protein/P96110.4?report=genbank&log$=protalign&blast_rank=1&RID=XGXGH267014) |  |
|  |  | *gdhA2* | D3M96_12545 | GdhA2 | NADP-specific glutamate dehydrogenase | *E. coli* K12 (71/82) | | [P00370.1](https://www.ncbi.nlm.nih.gov/protein/P00370.1?report=genbank&log$=protalign&blast_rank=3&RID=XGXGH267014) |  |
|  |  | *guaA1* | D3M96_09105 | GuaA1 | GMP synthase [glutamine-hydrolyzing] | *B. petrii* DSM 12804 (83/90) | | [A9IKK0.1](https://www.ncbi.nlm.nih.gov/protein/A9IKK0.1?report=genbank&log$=protalign&blast_rank=1&RID=XGXGH267014) |  |
|  |  | *guaA2* | D3M96_11560 | GuaA2 | GMP synthase [glutamine-hydrolyzing] | *Streptococcus mutans* UA159 (33/52) | | [Q8DU81.1](https://www.ncbi.nlm.nih.gov/protein/Q8DU81.1?report=genbank&log$=protalign&blast_rank=1&RID=XGXGH267014) |  |
|  |  | *guaA3* | D3M96_14965 | GuaA3 | GMP synthase [glutamine-hydrolyzing] | *Parabacteroides distasonis* ATCC 8503 (34/45) | | [A6LD04.1](https://www.ncbi.nlm.nih.gov/protein/A6LD04.1?report=genbank&log$=protalign&blast_rank=1&RID=XGXGH267014) |  |
|  |  | *glmS* | D3M96_18780 | GlmS | Glutamine--fructose-6-phosphate aminotransferase [isomerizing] | *B. bronchiseptica* RB50 (83/92) | | [Q7WE36.3](https://www.ncbi.nlm.nih.gov/protein/Q7WE36.3?report=genbank&log$=protalign&blast_rank=1&RID=XGXGH267014) |  |
|  |  | *bauA* | D3M96_15060 | BauA | Beta-alanine--pyruvate aminotransferase | *P. aeruginosa* PAO1 (74/85) | | [Q9I700.1](https://www.ncbi.nlm.nih.gov/protein/Q9I700.1?report=genbank&log$=protalign&blast_rank=1&RID=XGXGH267014) |  |
|  |  | *doeD* | D3M96_01785 | DoeD | Diaminobutyrate--2-oxoglutarate transaminase | *Halomonas elongata* 1H9 (33/52) | | [E1V7V7.1](https://www.ncbi.nlm.nih.gov/protein/E1V7V7.1?report=genbank&log$=protalign&blast_rank=5&RID=XGXGH267014) |  |
|  |  | *alaC* | D3M96_06795 | AlaC | Glutamate-pyruvate aminotransferase | *E. coli* K12 (80/90) | | [P77434.1](https://www.ncbi.nlm.nih.gov/protein/P77434.1?report=genbank&log$=protalign&blast_rank=1&RID=XGXGH267014) |  |
|  |  | *dag* | D3M96_18645 | Dag | N-acyl-D-glutamate deacylase | *Achromobacter xylosoxydans* A-6 (71/82) | | [P94211.1](https://www.ncbi.nlm.nih.gov/protein/P94211.1?report=genbank&log$=protalign&blast_rank=1&RID=XGXGH267014) |  |
|  |  | *alaA* | D3M96_12920 | AlaA | Glutamate-pyruvate aminotransferase AlaA | *E. coli* O6:H1 (59/77) | | [P0A960.1](https://www.ncbi.nlm.nih.gov/protein/P0A960.1?report=genbank&log$=protalign&blast_rank=1&RID=XGXGH267014) |  |
|  |  | *yerD* | D3M96_08220 | YerD | Glutamate synthase large subunit-like protein YerD | *B. subtilis* 168 (40/54) | | [O34849.1](https://www.ncbi.nlm.nih.gov/protein/O34849.1?report=genbank&log$=protalign&blast_rank=1&RID=XGXGH267014) |  |
|  |  | *astE* | D3M96_10315 | AstE | Succinylglutamate desuccinylase | *Paraburkholderia xenovorans* LB400 (31/48) | | Q141D1.1 |  |
|  |  |  |  |  |  |  | |  |  |
| Glutamine | Transport | *glnH* | D3M96_04815 | GlnH | Glutamine ABC transporter substrate-binding protein GlnH | *E. coli* O157:H7 (56/72) | | [P0AEQ5.1](https://www.ncbi.nlm.nih.gov/protein/P0AEQ5.1?report=genbank&log$=protalign&blast_rank=1&RID=XGXGH267014) |  |
|  |  | *glnP* | D3M96_04820 | GlnP | Glutamine transport system permease protein GlnP | *E. coli* O157:H7 (60/79) | | [P0AEQ8.1](https://www.ncbi.nlm.nih.gov/protein/P0AEQ8.1?report=genbank&log$=protalign&blast_rank=1&RID=XGXGH267014) |  |
|  |  | *glnQ* | D3M96_04825 | GlnQ | Glutamine ABC transporter ATP-binding protein GlnQ | *E. coli K12 (69/82)* | | [P10346.1](https://www.ncbi.nlm.nih.gov/protein/P10346.1?report=genbank&log$=protalign&blast_rank=1&RID=XGXGH267014) |  |
|  |  | *bztD* | D3M96_16960 | BztD | Glutamate/glutamine/aspartate/asparagine transport ATP-binding protein | *Rhodobacter capsulatus* SB1003 (73/87) | | [Q52666.2](https://www.ncbi.nlm.nih.gov/protein/Q52666.2?report=genbank&log$=protalign&blast_rank=3&RID=XFH96WDS014) |  |
|  |  | *bztC* | D3M96_16965 | BztC | Glutamate/glutamine/aspartate/asparagine transport system permease protein BztC | *R. capsulatus* SB1003 (40/53) | | [Q52665.1](https://www.ncbi.nlm.nih.gov/protein/Q52665.1?report=genbank&log$=protalign&blast_rank=3&RID=XFH96WDS014) |  |
|  |  | *bztB* | D3M96_16970 | BztB | Glutamate/glutamine/aspartate/asparagine transport system permease protein BztB | *R. capsulatus* SB1003 (42/58) | | [Q52664.1](https://www.ncbi.nlm.nih.gov/protein/Q52664.1?report=genbank&log$=protalign&blast_rank=3&RID=XFH96WDS014) |  |
|  |  | *bztA* | D3M96_16975 | BztA | Glutamate/glutamine/aspartate/asparagine-binding protein BztA | *R. capsulatus* SB1003 (54/68) | | [Q52663.2](https://www.ncbi.nlm.nih.gov/protein/Q52663.2?report=genbank&log$=protalign&blast_rank=4&RID=XFH96WDS014) |  |
|  | Biosynthesis | *glnE* | D3M96_12560 | GlnE | Bifunctional glutamine synthetase | *B. bronchiseptica* RB50 (62/73) | | [Q7WHH4.1](https://www.ncbi.nlm.nih.gov/protein/Q7WHH4.1?report=genbank&log$=protalign&blast_rank=1&RID=XJUV7JJ0014) |  |
| Proline | Transport | *putP* | D3M96_01750 | PutP | High-affinity proline transporter PutP | *B. subtilis* 168 (23/44) | | [P94392.3](https://www.ncbi.nlm.nih.gov/protein/P94392.3?report=genbank&log$=protalign&blast_rank=2&RID=XGXGH267014) |  |
|  | Biosynthesis | *proA* | D3M96_13485 | ProA | Glutamate-5-semialdehyde dehydrogenase | *Bordetella avium* 197N (69/81) | | [Q2KXE0.1](https://www.ncbi.nlm.nih.gov/protein/Q2KXE0.1?report=genbank&log$=protalign&blast_rank=1&RID=XGXGH267014) |  |
|  |  | *proB* | D3M96_16910 | ProB | Glutamate 5-kinase | *B. avium* 197N (74/85) | | [Q2L067.1](https://www.ncbi.nlm.nih.gov/protein/Q2L067.1?report=genbank&log$=protalign&blast_rank=1&RID=XGXGH267014) |  |
|  |  | *proC* | D3M96_10620 | ProC | Pyrroline-5-carboxylate reductase | *P. aeruginosa* PAO1 (45/60) | | [P22008.2](https://www.ncbi.nlm.nih.gov/protein/P22008.2?report=genbank&log$=protalign&blast_rank=1&RID=XGXGH267014) |  |
|  |  | *argF* | D3M96_08585 | ArgF | Ornithine carbamoyltransferase | *B. pertussis* Tahoma I (84/91) | | [Q7VTJ8.1](https://www.ncbi.nlm.nih.gov/protein/Q7VTJ8.1?report=genbank&log$=protalign&blast_rank=1&RID=XGXGH267014) |  |
| Glycine betaine/choline | Transport | *ousV* | D3M96_13435 | OusV | Glycine betaine/choline transport system ATP-binding protein OusV | *Dickeya dadantii* 3937 (49/69) | | [E0SCY1.1](https://www.ncbi.nlm.nih.gov/protein/E0SCY1.1?report=genbank&log$=protalign&blast_rank=1&RID=XGXGH267014) |  |
|  |  | *ousW* | D3M96_13440 | OusW | Glycine betaine/choline transport system permease protein OusW | *D. dadantii* 3937 (47/66) | | [E0SCY2.1](https://www.ncbi.nlm.nih.gov/protein/E0SCY2.1?report=genbank&log$=protalign&blast_rank=1&RID=XGXGH267014) |  |
| Alanine | Biosynthesis | *iscS* | D3M96_08980 | IscS | IscS subfamily cysteine desulfurase | *Methylococcus capsulatus* ATCC 33009 (70/85) | | [Q60C64.1](https://www.ncbi.nlm.nih.gov/protein/Q60C64.1?report=genbank&log$=protalign&blast_rank=1&RID=XJU9EEZZ014) |  |
|  |  | *sufS* | D3M96_10015 | SufS | Cysteine desulfurase | *Salmonella arizonae* RSK2980 (52/68) | | [A9MEP3.1](https://www.ncbi.nlm.nih.gov/protein/A9MEP3.1?report=genbank&log$=protalign&blast_rank=1&RID=XJU9EEZZ014) |  |
|  |  | *sufE* | D3M96_09215 | SufE | Cysteine desulfuration protein SufE | *Pectobacterium atrosepticum* SCRI 1043 (37/56) | | [Q6D624.1](https://www.ncbi.nlm.nih.gov/protein/Q6D624.1?report=genbank&log$=protalign&blast_rank=4&RID=XJU9EEZZ014) |  |
